# Distinguishing recrudescence from reinfection in lymphatic filariasis

**DOI:** 10.1016/j.ebiom.2024.105188

**Published:** 2024-06-07

**Authors:** Young-Jun Choi, Kerstin Fischer, Aboulaye Méité, Benjamin G. Koudou, Peter U. Fischer, Makedonka Mitreva

**Affiliations:** a Infectious Diseases Division, Department of Medicine, Washington University School of Medicine, St. Louis, MO, USA; b Programme National de la Lutte Contre la Schistosomiase, Les Geohelminthiases et la Filariose Lymphatique, Abidjan, Côte d’Ivoire; c Centre Suisse de Recherche Scientifique en Côte d’Ivoire, Abidjan, Côte d’Ivoire; d Université Nangui Abrogoua, Abidjan, Côte d’Ivoire; e Department of Genetics, Washington University School of Medicine, St. Louis, MO, USA; f McDonnell Genome Institute, Washington University in St. Louis, St. Louis, MO, USA

**Keywords:** *Wuchereria bancrofti*, Microfilariae, Sibship inference, Whole-genome amplification, Exome capture, Genomic epidemiology

## Abstract

**Background:**

The Global Program to Eliminate Lymphatic Filariasis (GPELF) is the largest public health program based on mass drug administration (MDA). Despite decades of MDA, ongoing transmission in some countries remains a challenge. To optimise interventions, it is critical to differentiate between recrudescence and new infections. Since adult filariae are inaccessible in humans, deriving a method that relies on the offspring microfilariae (mf) is necessary.

**Methods:**

We developed a genome amplification and kinship analysis-based approach using *Brugia malayi* samples from gerbils, and applied it to analyse *Wuchereria bancrofti* mf from humans in Côte d’Ivoire. We examined the pre-treatment genetic diversity in 269 mf collected from 18 participants, and further analysed 1-year post-treatment samples of 74 mf from 4 participants. Hemizygosity of the male X-chromosome allowed for direct inference of haplotypes, facilitating robust maternal parentage inference. To enrich parasite DNA from samples contaminated with host DNA, a whole-exome capture panel was created for *W. bancrofti*.

**Findings:**

By reconstructing and temporally tracking sibling relationships across pre- and post-treatment samples, we differentiated between new and established maternal families, suggesting reinfection in one participant and recrudescence in three participants. The estimated number of reproductively active adult females ranged between 3 and 11 in the studied participants. Population structure analysis revealed genetically distinct parasites in Côte d’Ivoire compared to samples from other countries. Exome capture identified protein-coding variants with ∼95% genotype concordance rate.

**Interpretation:**

We have generated resources to facilitate the development of molecular genetic tools that can estimate adult worm burdens and monitor parasite populations, thus providing essential information for the successful implementation of GPELF.

**Funding:**

This work was financially supported by the 10.13039/100000865Bill and Melinda Gates Foundation (https://www.gatesfoundation.org) under grant OPP1201530 (Co-PIs PUF & Gary J. Weil). *B. malayi* parasite material was generated with support of the Foundation for Barnes Jewish Hospital (PUF). In addition, the development of computational methods was supported by the 10.13039/100000002National Institutes of Health under grants AI144161 (MM) and AI146353 (MM). The funders had no role in the study design, data collection and analysis, decision to publish, or preparation of the manuscript.

**Research in context**

**Evidence before this study**

A PubMed search conducted on November 7, 2023, using the search terms “filarial parasite AND (genome OR diagnosis OR MDA OR epidemiology)” without date restrictions, indicated that the currently available diagnostic methods for lymphatic filariasis are insufficient for accurately estimating the number of breeding adult worms or differentiating between recrudescence and reinfection following treatment. Previous studies suggested that genetics-based relatedness analysis of the offspring parasites can provide insights into the breeding adult population within the host. However, previous studies failed to obtain whole-nuclear genome data, limiting the analysis of mf, which remained a challenge due to the low DNA yield. A selective whole-genome amplification method resulted in variable and partial coverage of the nuclear genome. These studies suggested that with the development of a reliable genotyping and kinship analysis-based approach, it would be feasible to genetically track and quantitatively monitor (inaccessible) adult worms throughout the treatment process in clinical studies by using genetic information from mf collected from human infections.

**Added value of this study**

We developed a robust single mf sample preparation, genome amplification, and sequencing pipeline that achieves over 90% coverage of the nuclear genome. Using these high-quality genotypic data, we developed a sibship inference method that utilises the X-chromosome haplotype diversity of male offspring parasites in addition to autosomal genetic variants. This maternal parentage inference approach was crucial for the successful analysis of clinical isolates of *Wuchereria bancrofti*, which exhibited low mitochondrial genetic diversity, rendering them unsuitable for kinship inference. We estimated the number female breeders, genetically tracked these maternal lineages through treatment, and distinguished between recrudescence and reinfection, using mf samples from an open-label randomised controlled drug trial in Côte d’Ivoire. Our genomic approach also revealed parasites that are genetically distinct from previously sequenced populations, suggesting that the expansion of the databases to include geographically diverse samples will allow for the spatial tracking of parasite movement associated with host or vector migration. Furthermore, we demonstrated the use of a targeted sequencing approach for genotyping samples with a higher rate of host contamination, which will aid in developing field-deployable tools for estimating worm burdens and monitoring parasite populations.

**Implications of all the available evidence**

Differentiating recrudescence due to treatment failure and new infection from ongoing transmission is key to understanding the cause of rebound infections and optimising interventions for permanent elimination. Continued advancements of these genomics-based approaches will provide essential information for the successful implementation of lymphatic filariasis elimination programs.

## Introduction

Population genomic approaches are continually transforming the field of infectious disease by enabling rigorous examination of epidemiological processes such as pathogen migration, strain divergence, and selection.^1^^,^^2^ For parasitic infections, genomic surveillance through spatiotemporal analysis of the distribution of genetic variation within and between hosts and geographic regions can uncover transmission dynamics, hybridisation/introgression events, use of reservoir hosts, and the impact of intervention strategies, such as mass drug administration (MDA), on parasite populations.^3^, ^4^, ^5^, ^6^ Lymphatic filariasis (LF) is a widespread mosquito-borne parasitic disease targeted for global elimination by the World Health Organization,^7^ and has remained relatively untouched by the advances in genomic approaches compared to other pathogen groups, despite its global health significance.^7^^,^^8^ One reason that progress has been difficult is that the adult stages of the LF-causing nematodes (*W. bancrofti*, *Brugia malayi* and *Brugia timori*) cannot be easily isolated from infected humans, since they reside in the lymphatics. The adult stage causes chronic diseases such as lymphedema and elephantiasis or hydrocele in men. Adult worms produce microfilariae (mf) that circulate in the blood and are readily available for genomic characterisation. However, the amount of DNA obtained from a single mf is insufficient for genome sequencing. Such technical obstacles have restricted population-scale genomic analyses of field isolates in humans.^9^ Earlier studies also depended on laboratory (animal) passages to obtain developmental stages (adults or L3s) that yield higher quantities of DNA.^10^^,^^11^ However, these experimental passages can lead to a loss of genetic diversity and unintended changes in allele frequencies due to selection or genetic drift.

Genomic analysis of individual mf using Next Generation Sequencing (NGS) can, however, be achieved through DNA amplification. A 2019 study applied selective whole-genome amplification (SWGA) to *W. bancrofti*.^9^ The SWGA approach utilises isothermal amplification based on the highly processive Phi29 DNA polymerase, similar to the conventional whole-genome amplification (WGA) method. However, SWGA differs from WGA in that it uses primers based on nucleotide sequence motifs that are prevalent in the target genome (parasite) but infrequent in the background genome (host), rather than using random hexamers. This strategy selectively amplifies the target genome from samples in which it originally represented a minor fraction of the total DNA. While SWGA has been employed in genomic studies of bacteria and *Plasmodium* species from clinical samples,^12^ the efficiency and reliability of the method have not been systematically tested for multicellular eukaryotic pathogens, such as filarial nematodes, which possess relatively large and complex genomes.

Similar to other helminth infections where the adult-stage parasites cannot be easily obtained from human hosts, the infection burden of filarial parasites is measured indirectly by counting larvae or through serologic means. These methods do not offer sufficient data to accurately estimate the number of reproductively active adult parasites or to quantitatively track changes in the adult worm population within the host. As a result, distinguishing between recrudescence (poor drug response leading to persistent infection) and new infections (ongoing transmission) after treatment using these indirect methods is challenging. Nonetheless, understanding the cause of rebound infections is crucial for permanent elimination. Within an area that passed the WHO recommended transmission assessment surveys and stopped MDA, it is possible that transmission may resume without the introduction of new infections.^13^^,^^14^ Reasons for this could include: (i) hidden active infections because of non-compliance to MDA, (ii) spontaneous resumption of mf production because treatment does not kill all adult worms, which have a long life expectancy, or (iii) reinfection from animal reservoirs (in the case of *B. malayi*). Resumption of effective MDA with the same or a more efficacious drug combination may solve the problem in these cases.^15^ In contrast, transmission may also resume because of the introduction of parasites from other regions by infected mosquitoes that may fly or drift long distances, or because of immigrants from LF endemic areas.^16^ For these cases, resumption of MDA in the entire implementation unit may not be needed if reintroduction of parasites can be prevented.

Genetic analysis of offspring parasites allows for the inference of adult parasite populations that are not directly accessible for examination.^17^ Through kinship analysis, one can reconstruct family structures and estimate the number of breeding adults. By monitoring these families over time, insights into adult worm survival and fecundity can be gained. However, interpreting offspring genetic data requires careful analysis due to the complexity of parasite biology. For instance, the analysis can be influenced by polyandry,^10^^,^^18^ density-dependent fecundity,^19^ and sampling biases resulting from reproductive skew among the breeders.^20^

Here, we demonstrate that by analysing the genetic variation in mf before and after drug treatment, we can infer the survival/fecundity of adult worms. This analysis enables the identification of parasites (and their genotypes) that respond differently to treatment. Such identification is vital when investigating the role of parasite genetics in treatment outcomes, such as suboptimal drug responses. This advancement marks a significant and crucial step towards genomic epidemiology and surveillance, which will be invaluable in controlling filariasis. Building upon this, we have created *W. bancrofti* exon capture probes to mitigate the problem of host DNA contamination and aid in the development of genetic tools for estimating worm burdens and monitoring parasite population dynamics during MDA.

## Methods

### Ethics

*W. bancrofti* mf obtained from infected participants before and after treatment with ivermectin, diethylcarbamazine, and albendazole (IDA) triple-drug therapy were collected during an open-label randomised controlled trial that was previously published.^21^ As published previously, the trial was registered at Clinicaltrials.gov (NCT02974049) and received approval by the University Hospitals of Cleveland Medical Center Institutional Review board and the Comité National d’Ethique et de la Recherche of Côte d’Ivoire.^21^ Genome sequencing of the mf from de-identified human samples performed in the present study received a full waiver of HIPAA Authorization (IRB ID #: 201103313), and non-human subjects determination (IRB ID #: 201712119) by the Washington University in St. Louis Institutional Review Board (DHHS Federalwide Assurance #FWA00002284). *B. malayi* samples were obtained from experimentally infected animals. All animal experiments were carried out under protocols approved by Washington University School of Medicine (20-0503) Institutional Animal Care and Use Committees (IACUC). All housing and care of laboratory animals conformed to the National Institutes of Health (NIH) Guide for the Care and Use of Laboratory Animals in Research (see 18-F22). Euthanasia was accomplished by CO_2_ inhalation.

### Parasite material—*B. malayi*

Adult *B. malayi* of the TRS strain^22^ were produced in gerbils as described previously.^23^ To generate DNA for experiments comparing different whole-genome amplification (WGA) methods, adult males were collected and their DNA was isolated using the whole worm. Sixteen individual females were collected from 2 gerbils, washed in PBS, and cultured overnight in RPMI medium at 37 °C. Thereafter, the apical end of the female (proximal to the genital opening) was removed and stored for isolation of maternal somatic DNA while minimizing DNA contamination from embryonic material. Microfilariae, which had been released into the RPMI medium, were collected from each female and stored at −20 °C. After whole-genome sequencing analysis of the *B. malayi* females, 2 female worms displaying high levels of heterozygosity were selected, and 40 of their F_1_ progeny mf that had been collected as described above were selected for DNA isolation.

### Parasite material—*W. bancrofti*

Blood samples of participants who were *W. bancrofti* mf-positive were collected before and after treatment during a clinical trial in southeastern Côte d’Ivoire, as previously described (Supplementary Tables S1 and S2).^21^^,^^24^ De-identified venous blood samples were collected at night using EDTA coated vacutainers and blood cells (red cells and buffy coat) that contained mf were stored frozen at −20 °C and shipped frozen to Washington University School of Medicine in St. Louis where the samples were stored at −80 °C. For isolation of individual mf, samples were thawed on ice. Depending on the mf concentration in the blood, 20–100 μl of blood was mixed with 900 μl PBS. Then, 200 μl of this solution was pipetted onto a slide and single mf were picked under a Primostar dissecting microscope (Zeiss, Jena, Germany) at 4-fold magnification. The mf were collected in a 1.5 ml tube containing 200 μl fresh PBS. These mf were then placed onto a slide and single mf were picked and collected in a 1.5 ml tube containing fresh PBS. These washing steps were repeated 3–5 times, depending on the initial volume of blood. In the final step, single mf were picked from the slide using a micropipettor (10 μl volume), transferred individually to 0.2 ml microfuge tubes, and were either used for DNA extraction or stored at −20 °C until further use.

### DNA isolation, whole-genome amplification and sequencing

The DNeasy Blood and Tissue kit (Qiagen) was used for DNA isolation from adult *B. malayi* worms, followed by ethanol precipitation. For DNA isolation from single mf, we used the CGP protocol^25^ with modifications: 25 μl of lysis buffer (950 μl nuclease-free water, 30 μl 3 M Tris–HCL, 5 μl NP-40, 5 μl Tween-20, and 10 μl proteinase K) was added to each tube containing one mf. The samples were then incubated at 55 °C for minimum of 2 h, followed by a 20 min 85 °C proteinase K inactivation, and finally stored at 4 °C. Successful DNA extraction was confirmed by qPCR using HhaI and LDR primers for *B. malayi* and *W. bancrofti*, respectively (Supplementary Table S3).^26^^,^^27^ Positive samples were then amplified using the Ready-To-Go GenomiPhi V3 DNA Amplification kit (Cytiva, Marlborough, MA) according to the manufacturer’s recommendations. After WGA, the sample was diluted 1:10, and the presence of parasite DNA was confirmed by qPCR. For the quantification of human DNA, we used primers that amplify a section of Chromosome I.^10^

Selective whole-genome amplification (SWGA) was carried out as previously described^28^ with slight modifications. Primers were designed using the SWGA primer design toolkit, swga v0.4.4,^28^ with *B. malayi* nuclear, mitochondria, and the *Wolbachia* endosymbiont genomes (GenBank Accession: GCA_000002995.5, NC_004298.1, and AE017321.1)^29^ as the target and the human nuclear and mitochondria genomes (GenBank Accession: GCA_000001405.27)^30^ as the background. Following the guidelines provided by the authors, the maximum Gini coefficient parameter was set to 0.6, and up to 5 million primer sets were checked for compatibility, with a maximum set size of 10 primers. Sets with the lowest mean binding site distance and the lowest Gini index in the target genome were selected for experimental testing. Additionally, a manually curated set was chosen for its improved primer sequence diversity, achieved by removing redundant overlapping primers (Supplementary Table S4). Reactions were performed in a volume of 50 μl using input DNA (<1 ng/μl), 2.5 μM total of SWGA primers, 1 × phi29 buffer (New England Biolabs, Ipswich, MA), 1 × bovine serum albumin (BSA), 1 mM dNTPs, and 30 units phi29 polymerase (New England Biolabs). The amplification conditions were 35 °C for 5 min, 34 °C for 10 min, 33 °C for 15 min, 32 °C for 20 min, 31 °C for 30 min, 30 °C for 16 h, followed by deactivation of the enzyme at 65 °C for 15 min. WGA was performed under the same conditions but in a 20 μl reaction volume. Successful amplification was confirmed by qPCR, as was performed for the WGA samples. Amplified samples were directly used for library construction. Kapa Hyper PCR-free library was generated from the DNA sample and sequenced on Illumina’s NovaSeq platform (2 × 150 bp paired-end reads) to ∼10 Gb per sample.

### Analytical processing of the reads, variant calling, population structure and kinship analysis

Sequencing reads were adapter/quality trimmed using trimmomatic v0.39 and were aligned against the combined reference assembly of the filarial worm nuclear, mitochondria, and the *Wolbachia* endosymbiont genomes (GenBank Accession: GCA_000002995.5, NC_004298.1, and AE017321.1 for *B. malayi*; GCA_005281725.1, JF775522.1, and GCA_002204235.2 for *W. bancrofti*)^9^^,^^29^ using BWA v0.7.17.^31^ For clinical mf samples, the human genome was also included for sample quality assessment. Duplicate reads were removed and single-nucleotide variants (SNPs) were called using GATK v4.2.2.^32^ The following set of quality filters were applied to obtain high-confidence genotype calls in GATK: QD < 2; QUAL < 30; SOR > 3; FS > 60; MQ < 40; MQRankSum < −12.5; ReadPosRankSum < −8; DP > (2 × median depth).^33^ Variants were annotated according to their genomic locations and predicted coding effects using SnpEff v5.0c.^34^ PLINK v1.9^35^ was used for computing inbreeding coefficients (*F*_IS_) to identify samples with excessive heterozygosity (likely indicative of sample contamination). Male mf were identified based on the hemizygosity of the X-chromosome, resulting in a reduction in the number of mapped bases to the X-chromosome relative to the number of mapped bases to autosomes. High confidence biallelic SNP loci (minor allele count >4 and genotype missing rate <0.05) were used for downstream analysis. Population structure analysis^36^ was performed using PCA in PLINK v1.9^35^ and ADMIXTURE^37^ with Pong.^38^ These analyses were conducted after removing closely related samples with AKT v0.3.3^39^ and after eliminating loci in high linkage disequilibrium using PLINK v1.9^35^ (--indep-pairwise 500 kb 1 0.2). Kinship analysis was conducted to identify maternal siblings sharing the same (unobserved) mother by utilizing both autosomal and X-linked allele-sharing patterns. We employed the KING --build command as the initial step in identifying sibling groups among mf. This --build algorithm connects first-degree relatives using autosomal SNPs, thereby identifying potential full sibling groups (referred to as KING families). Given that a maximum of two X-linked haplotypes (along with their recombinants) is expected to segregate among full sibling or maternal half-sibling males, we assessed KING families for consistency with this expectation. Families that did not align with this expectation were adjusted (either divided or merged) to conform to the maximum of two X-linked haplotypes. To begin this process, we identified distinct maternally inherited X-linked haplotypes using male mf. We constructed maximum-likelihood phylogenetic trees using X-linked SNPs with IQ-TREE v2.2.0, where the best-fit model was automatically selected by ModelFinder. TreeCluster (--method max) facilitated the grouping of leaves into repeatedly observed haplotypes by identifying clusters of sequences with substantial similarity (--threshold 0.025). Singleton sequences remaining after this clustering process represented either (i) a low-frequency haplotype sampled only once or (ii) a recombinant haplotype, given the expected uniqueness of recombinant haplotypes due to variability in meiotic recombination breakpoints within gametes. We aimed to minimize the probability of classifying these recombinants as distinct haplotypes in our X-linked haplotype count. To this end, only singleton sequences significantly dissimilar from all others (--threshold 0.085) were considered in our analysis as distinct haplotypes. Singleton sequences that did not meet this threshold were not used in the downstream inference process. If a KING family exhibited more than two X-linked haplotypes, suggesting the presence of multiple maternal lineages, it was subdivided into separate maternal sibling groups based on autosomal genetic relatedness. Hierarchical clustering (Ward's minimum variance method) was applied to an all-vs.-all kinship coefficient matrix to identify subgroups within a KING family that correspond to distinct maternal sibling sets. The granularity of these clusters was chosen to meet the X-linked haplotype count criteria while also aiming to reduce the overall number of maternal families post-separation, adhering to the principle of parsimony. When identical X-linked haplotypes were found across multiple KING families, these families were consolidated into maternal (half-sibling) groups, provided there was at least one dyad with a kinship coefficient expected from a second-degree relationship (ranging between 0.0884 and 0.177) or higher, and the newly formed family still complied with the X-linked haplotype count requirement. To visually represent the family structure, PCA was performed using autosomal SNPs in PLINK, followed by UMAP (Uniform Manifold Approximation and Projection) in R using the first 6 principal components which explained 50–60% of the total variance. The local PCA approach, implemented in the *lostruct* package in R^40^ (with a sliding window of 20 SNPs and a sum of squared values of the covariance matrix >50) was used to examine the X-chromosome haplotype structure. ChromoMap^41^ was employed for haplotype visualization. Repeatedly observed haplotypes that showed highly similar patterns of PC1 values along their entire lengths were considered non-recombinant ‘parental’ haplotypes. Haplotype switching events were then inferred in the remaining (recombinant) sequences. The mitochondrial haplotype network was constructed in PopART^42^ using the Minimum Spanning Network method.^43^

### *W. bancrofti* exome sequencing and performance evaluation

A previously published *W. bancrofti* genome assembly (GenBank Accession: GCA_005281725.1)^9^ was re-annotated using both *ab initio* and homology-based annotation pipelines. Repetitive elements were softmasked with RepeatMasker v4.0.9 using a species-specific repeat library created by RepeatModeler v1.0.11.^44^ *Ab initio* gene predictions were generated using BRAKER2 v2.1.0,^45^ which was trained using *B. malayi* protein sequences (PRJNA10729). MAKER v2.31.10^46^ was subsequently run to refine these gene models and reduce false-positive gene predictions. *W. bancrofti* mf cDNA library (GenBank Accession: SAMN00155125), RNA-seq transcripts^9^ assembled using StringTie v1.3.6,^47^ *B. malayi* (PRJNA10729) CDS sequences, and protein sequences from WormBase ParaSite Version 13^48^ (*Acanthocheilonema viteae* PRJEB1697, *B. malayi* PRJNA10729, *Dirofilaria immitis* PRJEB1797, *Litomosoides sigmodontis* PRJEB3075, *Loa loa* PRJNA246086, *Onchocerca volvulus* PRJEB513) were provided to MAKER as transcript/protein evidence. Gene predictions without supporting evidence were excluded with the exception of those encoding Pfam domains, as detected by InterProScan v5.19,^49^ to balance sensitivity and specificity.^46^^,^^50^ Homology-based gene predictions were generated in GeMoMa v1.9^51^ using *B. malayi* gene models (PRJNA10729), and were combined with the BRAKER/MAKER *ab initio* gene predictions. For gene loci where both homology-based and *ab initio* gene predictions were available, homology-based gene models were selected in the final gene build. The completeness of the resulting gene set was assessed using BUSCO v5.4.7 with Nematoda specific single-copy orthologs (OrthoDB v10).^52^

### Exome capture panel, sequencing and evaluation

We designed custom hybridization capture reagents (Twist Bioscience, South San Francisco, CA) targeting the exonic regions of the *W. bancrofti* genome. A panel of 120 bp tiling-probes was designed to cover CDS targets defined by the BRAKER/MAKER *ab initio* gene predictions (described above). Problematic repeat regions were empirically identified from *W. bancrofti* whole-genome sequencing data based on read mappability (>50% mapped bases with mapping quality <10). Probes that overlapped with these regions, as well as those that exhibited high sequence similarity to the human genome (hg38) were removed from the design, resulting in a set of 150,846 probes (Twist Bioscience Custom Design ID: TE-92630402). This probe set covered 89% of the 14.9 Mb protein-coding space of the updated gene build. Hybridization reactions (8-plex), Streptavidin beads binding and purification were performed following manufacturer's instructions. Post-capture library pools were sequenced on Illumina’s NovaSeq platform (2 × 150 bp paired-end reads). Sequencing reads were processed and variants were called as described above for the whole-genome data, but the maximum DP filtering (GATK) was not applied. BEDTools v2.30^53^ and Picard v2.26.2 (CollectHsMetrics and GenotypeConcordance)^32^ were used to compute coverage and variant call metrics.

### Statistics

We evaluated the sensitivity, false discovery rate, and genotype concordance between variant call sets using the Picard GenotypeConcordance tool,^32^ where one set is considered the “truth” (reference) and the other the “call” being evaluated for accuracy. Sensitivity was calculated by dividing the number of true positives by the total number of positives, representing the proportion of true variants (those present in the truth dataset) correctly identified in our call set. The false discovery rate was determined by dividing the number of false positives by the sum of the true positives and false positives, indicating the proportion of false positive calls out of the total calls made. Genotype concordance refers to the proportion of true positives for which the correct genotype was accurately identified. We subsampled sequencing reads to uniform sizes using samtools (--subsample)^54^ when variation in total sequencing depth per library (i.e., library size) was a possible confounder in the performance assessment of WGA and exome capture data. We performed Fisher's exact test for maternal families in each participant to calculate the probability of observing the post-treatment mf counts, given the pre-treatment distribution. *p* values were corrected for multiple testing using the Benjamini-Hochberg false discovery rate (FDR). The assumptions for Fisher's exact test were met because the mf collection from each individual was random and independent, and the counts were low, making it more appropriate than the chi-squared test, which relies on an approximation to a distribution. To assess whether the pre-treatment sampling of mf was sufficient to determine the total number of maternal sibling groups in a participant, we performed a rarefaction analysis with bootstrapping (999 replicates) in iNEXT^55^ and calculated the asymptotic estimates of the total maternal family counts with 95% confidence intervals. ADMIXTURE modelling was performed to study population structure.^37^ Because the approach uses a greedy hill-climbing optimization algorithm, we performed 100 ADMIXTURE runs from different initial random starting points and summarized the output by collecting similar solutions into ‘modes' using Pong, and assessed the stability of the solution.^36^^,^^38^

### Role of funders

The funders had no role in study design, data collection, data analysis, interpretation, or writing of the manuscript.

## Results

**Fig. 1:**
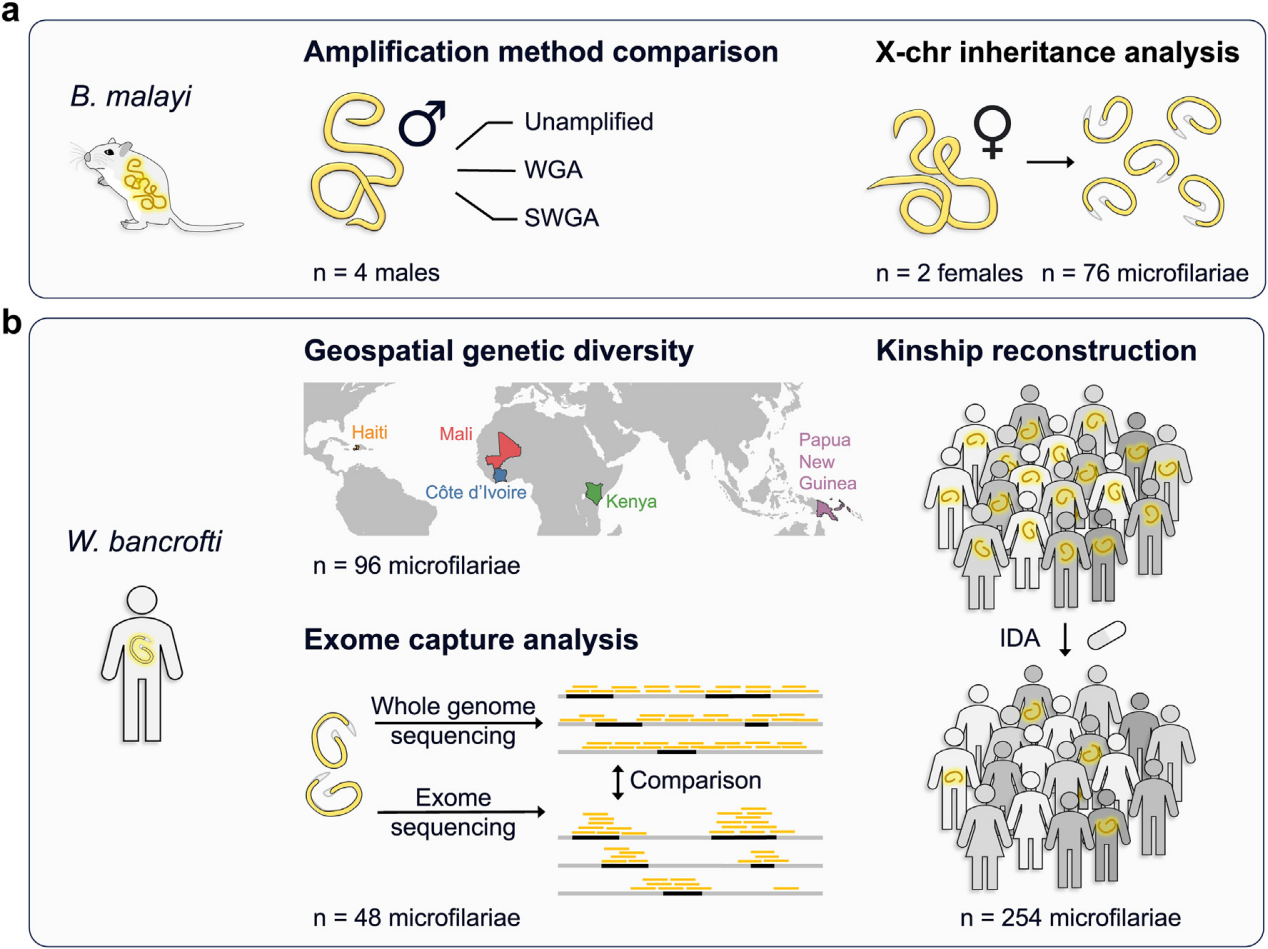
Overview of experiments and analysis. (a) *Brugia malayi* experiments. Adult worms collected from an experimental host (gerbils) and microfilariae of a known pedigree were used for methodology development. (b) *Wuchereria bancrofti* clinical isolates. Our genomics-based approach for analysing worm burden was applied to samples obtained from human infections in Côte d’Ivoire, which were also utilized to develop probes for exome sequencing.

The ultimate goals of the study were to 1) infer the number of reproductively active adult worms in participants infected with *W. bancrofti* based on the genome-wide genetic diversity among individual mf within a host, and 2) differentiate between new and persistent *W. bancrofti* infections by genetically tracking the survival and fecundity of unobserved adults throughout the treatment process. We undertook a step-wise approach to achieve these two goals. We took advantage of our *B. malayi*—gerbil infection model (Fig. 1a) to inform our *W. bancrofti* methodological and analytical approaches (Fig. 1b). A schematic representation of the overall study design, which summarizes the source and purpose of each sample set, is presented in Fig. 1. Initially, we evaluated WGA and SWGA methods to amplify filarial worm genomic DNA for sequencing-based genotyping. *B. malayi* adult male samples were used to (i) obtain sufficient DNA so that sequencing libraries could be generated from both amplified DNA and unamplified source DNA for direct comparison and (ii) avoid non-diploid genotypes from embryos, which would be present if gravid female samples were used. After establishing confidence in our methods, we proceeded to analyse a population of clinical field isolates of *W. bancrofti* collected from a recent open-label randomized controlled drug trial in Côte d’Ivoire.^21^ Furthermore, by leveraging the *B. malayi*—gerbil model, we produced mf with a known pedigree. This guided us to develop and validate our maternal sibship identification method, which utilizes X-chromosome genetic diversity. By longitudinally sampling post-treatment mf, we assessed the temporal shifts in parasite infra-populations a year after administering a single dose of a triple-drug treatment consisting of ivermectin, diethylcarbamazine, and albendazole (IDA).^21^ Lastly, we developed a whole-exome capture panel for *W. bancrofti*, designed to enrich parasite nuclear DNA from lower-quality samples that may contain host DNA contamination.

### Comparative analysis of genome coverage and genotyping reliability using different genome amplification methods for filarial DNA

**Fig. 2:**
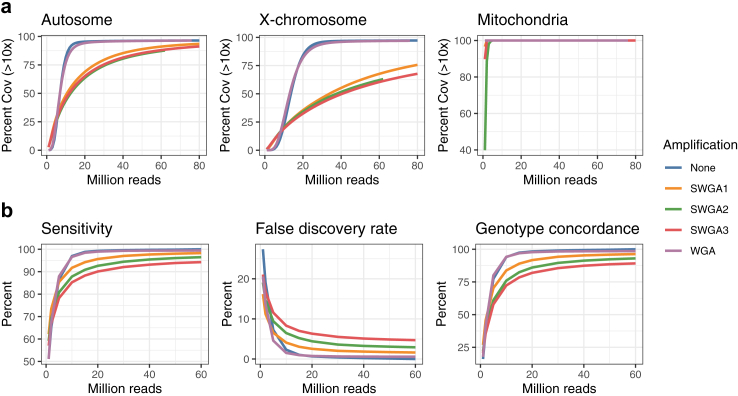
Performance assessment of genome amplification methods using *B. malayi* adult male worms. (a) Sequencing effort needed to achieve >10x coverage across the target genome. (b) Evaluation of variant call sets using the unamplified sample (60 million reads and ∼131 k autosomal SNPs) as the truth set.

Using DNA samples isolated from *B. malayi* adult males, we assessed the genome coverage bias and genotyping accuracy of WGA and SWGA methods relative to the unamplified DNA. SWGA primers were designed to minimize the distance between binding sites in the parasite genome and maximize the distance between binding sites in the host genome.^28^ Three SWGA primer sets were tested on single-worm samples (SWGA1: manually curated set to improve primer sequence diversity; SWGA2: algorithmically-generated set prioritized based on the binding site distance; SWGA3: algorithmically-generated set prioritized based on the binding site evenness) (Supplementary Table S4). For the WGA method, random primers were used. We estimated the change in genome coverage with varying sequencing effort by down-sampling each library (Fig. 2a). For a given library size (i.e., total bases sequenced), DNA samples without amplification achieved better genome coverage than amplified samples, and WGA was more effective in amplifying *B. malayi* genomic DNA compared to SWGA. Among the different SWGA primer sets, SWGA1 outperformed the others, although the differences were marginal. To achieve 90% coverage of the autosomes using WGA, sequencing of 15 million reads were required. To achieve a similar level of genome coverage using SWGA, 4 times more sequencing data (>60 million reads) were necessary. This implies that for samples containing up to ∼75% host DNA contamination, WGA would outperform SWGA (assuming that host and parasite DNA is amplified with equal efficiency in WGA and only parasite DNA is amplified in SWGA). Hemizygous sequences, such as the X chromosome in males, required ∼2 times more sequencing data compared to the diploid autosomes to achieve the same coverage. The mitochondrial genome was amplified effectively by both WGA and SWGA with minimal sequencing effort.

**Fig. 3:**
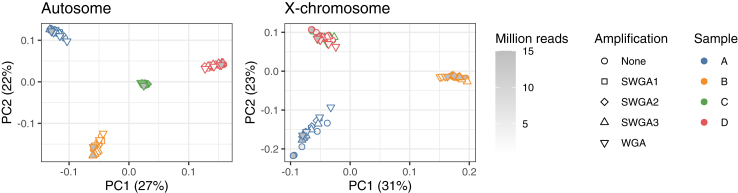
Principal component analysis of four individual *B. malayi* adult male worms. Genomic DNA samples were sequenced either without amplification or with WGA/SWGA. For each amplification group, variants were called after simulating 5 different library sizes (1, 2, 5, 10, 15 million reads). Each variant call set representing the unique combination of variables (worm, amplification method, and library size) were plotted as separate data points, and coloured according to the worm (samples A-D). Analysis was based on ∼251 k autosomal and ∼28 k X-linked SNPs. Adult male samples C and D had identical X-linked genotypes, suggesting that they are likely maternal siblings.

We quantified genotyping errors as a function of amplification method and library size (i.e., overall sequencing effort) (Fig. 2b) to better understand the level of technical artifacts from WGA/SWGA with respect to the biological genetic variability (Fig. 3). Using the unamplified DNA sample as the ground truth, we evaluated the sensitivity, false discovery rate, and genotype concordance of the variant call sets generated by both the WGA and the SWGA method. The decrease in genome coverage associated with amplification (Fig. 2a) resulted in a lower sensitivity for SNP detection (Fig. 2b). In addition, the number of false positive variants and discordant genotypes increased when DNA samples were amplified using WGA or SWGA. Our data also showed that despite the increase in genotyping errors, samples clustered in PCA by worm and not by amplification method or library size (Fig. 3), indicating that genome-wide SNP data generated using amplified DNA correctly captured the genetic relationship between individual worms. This in turn suggested that temporal changes in (infra)population structure associated with treatment could be studied using WGA or SWGA approach.

### Estimating the number of reproductively active adult parasites based on the genetic diversity of offspring microfilariae

**Fig. 4:**
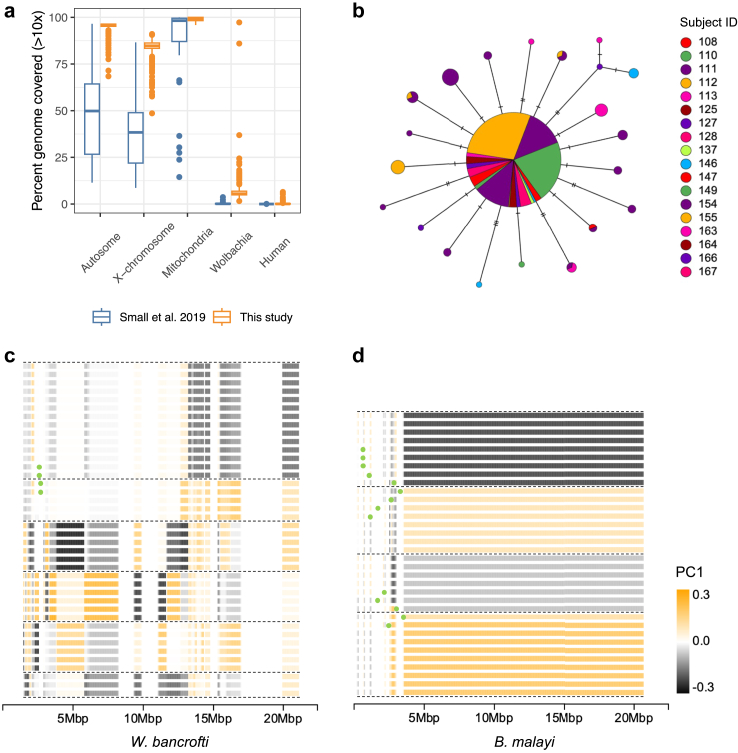
Analysis of mitochondrial and X-chromosomal genetic diversity using single microfilaria sequencing. (a) The improved genome coverage from single microfilaria sequencing using the optimized WGA protocol developed in this study, compared to previously published SWGA libraries by Small et al.^9^ (b) Mitochondrial haplotype network of 343 *W. bancrofti* mf collected from 18 participants in Côte d’Ivoire. Male X-chromosome haplotypes inferred using “local” principal component analysis in (c) *W. bancrofti* mf collected from participant 155 (n = 40), and (d) *B. malayi* mf collected from 2 adult females (n = 34). Horizontal lines represent individual haplotypes. PCA was performed along the length of chromosome (excluding the pseudoautosomal region) in sliding windows of 20 SNPs. A total of 1081 X-linked SNPs were used in *W. bancrofti* and 5491 in *B. malayi*, respectively. PC1 values were used to color each window. Recombination breakpoints were marked by green dots.

We sequenced a total of 343 *W. bancrofti* mf samples collected from the IDA trial in Côte d’Ivoire,^21^ including pre-treatment samples from 14 participants (n = 96) and pre- and 1-year post-treatment paired samples from 4 participants (n = 247). Guided by our *B. malayi* analysis which demonstrated that WGA achieved better genome coverage compared to SWGA, we used the WGA method to sequence individual *W. bancrofti* mf. Initial sequencing of the amplified DNA indicated that the level of host DNA contamination could be high and variable between samples (data not shown). Considering the 38-fold difference in genome size, even a small number of contaminating human cells can result in a significant number of human-derived reads in the final sequencing data. We therefore directed our efforts to improve the sample preparation method to minimize host cell contamination. Our approach (described in detail in the Methods section) included multiple washing steps and qPCR-based quality screening steps to ensure all samples have acceptable worm-to-human DNA ratios (9:1). On average, more than 95% of the autosome, 85% of the X-chromosome, and 99% of the mitochondrial genome were covered by at least 10x (Fig. 4a).

Because mitochondrial genomes (mtDNA) are maternally inherited, we first investigated whether we could estimate the number of adult female worms by analysing the mitochondrial genetic diversity among mf sampled from each human participant. Sequence comparisons of the 13.5 kb mtDNA across all samples revealed low genetic diversity. We observed extensive haplotype sharing between unrelated mf collected from different participants (Fig. 4b). These results indicate that mitochondrial data are not suitable for parentage inference in *W. bancrofti*. Like other filarial worms, *W. bancrofti* has an XY sex-determination system, and the inheritance pattern of the X-chromosome should allow the determination of maternal lineages using X-linked loci in male F_1_ progeny mf. Due to the hemizygous state of the male X-chromosome, haplotypes can be directly inferred from SNP data. By performing “local” principal component analysis (PCA) in sliding windows along the length of the chromosome, we could examine the long-range haplotype structure at the chromosomal level across a large number of mf samples (Fig. 4c and d). For instance, among the male mf collected from participant 155 (n = 40), we identified 6 major haplotypes, including 4 sequences representing recombinant haplotypes (Fig. 4c). Since a single diploid female worm can pass on 2 X-chromosome haplotypes (and their crossover recombinants) to the next generation, these results suggest that mf from participant 155 descended from 3 female worms.

To better understand the inheritance patterns of X-chromosome genetic diversity in filarial parasites and validate our interpretation of the data, we generated genotype data from samples with known familial relationships using experimentally tractable *B. malayi* worms maintained in gerbils (TRS strain). Due to the low expected genetic diversity of this inbred laboratory strain, we sequenced somatic DNA samples from 7 adult females to select 2 female worms with higher levels of X-chromosome heterozygosity (Supplementary Table S5). Subsequently, the F_1_ progeny mf (n = 76) of these females were genotyped. Using the sliding window PCA approach, we determined the X-chromosome haplotype structure among the male mf (n = 34). As expected, 4 major haplotype groups were identified, including their recombinant forms, consistent with 2 maternal lineages (Fig. 4d). These data also suggested that meiotic recombination predominantly occurred at the distal ends of the X-chromosome (proximal to the telomeres) in both *W. bancrofti* and *B. malayi*. Analysis of the haplotype structure, excluding these regions of high recombination rates, can provide complementary information helpful for determining the number of unique haplotype groups that are informative for maternal parentage inference.

### Discriminating persistent vs. new *W. bancrofti* infections based on kinship analysis of microfilariae before and after treatment

**Fig. 5:**
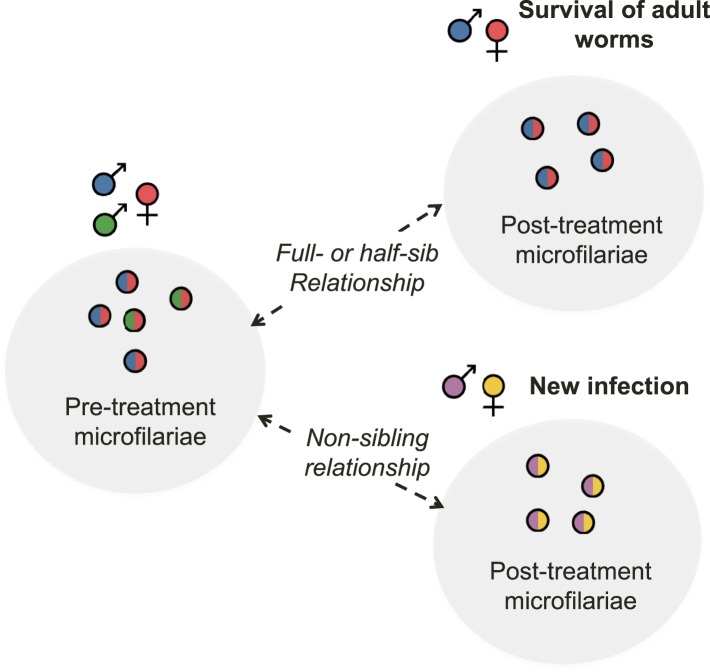
Schematics for the identification of treatment failure and reinfection based on kinship analysis of offspring worms. The presence of sibling relationships across pre- and post-treatment microfilariae indicates the survival of parental adult worms, while the absence of such relationships indicates a newly acquired infection.

**Fig. 6:**
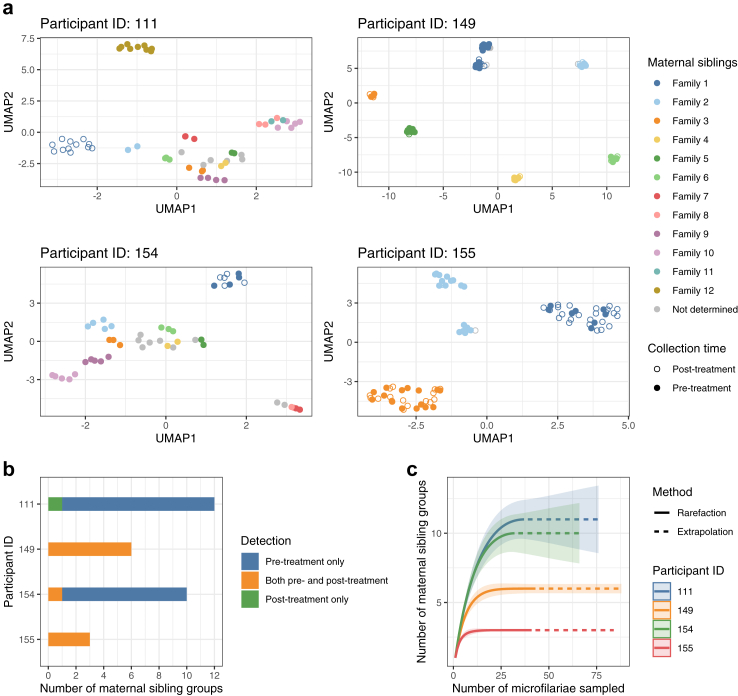
Monitoring worm burden based on the infra-population genetic diversity in *W. bancrofti*. (a) Maternal sibship relationship among *W. bancrofti* microfilariae. Kinship was inferred across pre- and post-treatment samples in each participant using autosomal and X-linked SNPs. UMAP was performed using the first six principle components of autosomal PCA. (b) Estimation of the minimum number of reproductively active female breeders based on the mf maternal sibling groups. (c) Rarefaction and extrapolation sampling curves for the number of pre-treatment maternal sibling groups. Bootstrapping confidence intervals (95%) were shown as shaded areas around the lines.

Next, we examined changes in the parasite infra-population, and investigated if the post-treatment populations likely represent a new or persistent infection (Fig. 5). Pairwise fixation index (*F*_*ST*_) analysis (Supplementary Table S6) indicated that there were varying degrees of genetic shift in parasite populations in each participant at 1-year post-treatment, ranging from a small change (e.g., participant 149) to a larger change (e.g., participant 111). We reconstructed familial relationships among individual mf (Fig. 6a). We first identified potential full sibling groups (referred to as KING families in Supplementary Fig. S1) using both male and female mf with KING,^56^ a family relationship inference tool. We then inferred maternal sibling groups (Supplementary Table S7) by dividing or merging the KING families based on the segregation pattern of X-linked haplotypes identified in male mf (Supplementary Fig. S2). The merging of KING families into maternal half-sibling groups was also guided by kinship coefficient values (Supplementary Table S8) expected to range between 0.0884 and 0.177 for second-degree relationships. The analysis revealed persistent infections in participants 149, 154, and 155, where individual mf from both pre- and post-treatment populations belonged to the same maternal family. In contrast, for participant 111, none of the post-treatment mf showed a maternal sibling relationship with any of the pre-treatment mf, indicating a new infection (Fisher's exact test with FDR correction, *p* = 2.3 × 10^11^; Supplementary Table S9). Based on the maternal sibling groups, we estimated the minimum number of reproductively active adult females to range from 3 in participant 155 to 11 in participant 111 (Fig. 6b). Because our ability to detect sibling families depended on the depth of mf sampling for each participant, we constructed rarefaction and extrapolation sampling curves with 95% confidence intervals using bootstrapping (Fig. 6c) and calculated the asymptotic estimates of the total maternal family counts in pre-treatment samples (Table 1).^57^ The upper 95% confidence limit was higher than the number of observed sibling families in participants 111, 149, and 154, albeit to varying degrees, indicating deeper sampling of mf may identity additional sibling groups.

**Table 1:** Asymptotic estimates of the total number of pre-treatment maternal sibling families of *W. bancrofti*.

Participant ID	Observed	Estimator	95% confidence limits	
			Lower	Upper
111	11.0	11.0	11.0	15.2
149	6.0	6.0	6.0	6.2
154	10.0	10.0	10.0	13.5
155	3.0	3.0	3.0	3.0

### *W. bancrofti* genetic diversity across geographic regions

**Fig. 7:**
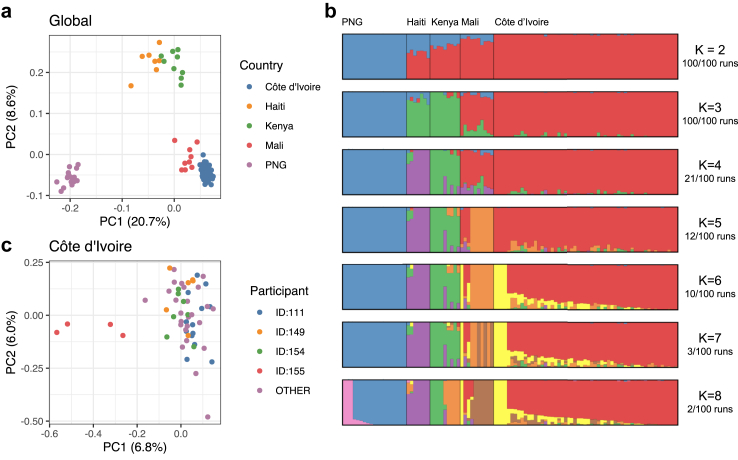
Principal component analysis and ADMIXTURE modelling of *W. bancrofti* genetic diversity. (a and b) Publicly available genomic data for *W. bancrofti*^9^^,^^10^ were included to contextualize the genetic diversity of the samples collected from Côte d’Ivoire. Analyses were based on 3334 LD-pruned autosomal SNPs and 100 unrelated samples. The major mode from 100 ADMIXTURE runs was displayed, and the stability of the solution was reported. (c) Principal component analysis based on 2380 LD-pruned autosomal SNPs and 55 unrelated Côte d’Ivoire samples.

We examined the population structure of *W. bancrofti* in our study site in Côte d’Ivoire to investigate the possibility of identifying migrant parasites from ancestrally diverse populations. In addition to the 4 participants from whom we collected paired samples both pre-treatment and 1-year post-treatment (n = 254), we generated pre-treatment mf genomic data (n = 96) collected from 14 additional participants from Côte d’Ivoire. To put the genetic diversity of these Côte d’Ivoire samples into context, we conducted principal component analysis (PCA) and ADMIXTURE modelling, incorporating previously published samples from Haiti, Kenya, Mali, and PNG (Fig. 7a and b).^9^^,^^10^ Closely related samples and variants in strong linkage disequilibrium were excluded from these analyses. The Côte d’Ivoire samples formed a distinct cluster in the PCA space with variance components more similar to samples from Mali than to those from other countries, in line with the relative geographical proximity between the two countries (Fig. 7a). Genetic clustering based on the admixture model indicated that the data support up to three highly differentiated populations (Fig. 7b). Under this model, our Côte d’Ivoire samples displayed relatively homogeneous genetic ancestries. When PCA was performed using samples from Côte d’Ivoire only, samples from participant 155 were separated from all other samples collected from 17 other participants along the first principal component (Fig. 7c), suggesting a possible genetic divergence from the local parasite population at the study site in Côte d’Ivoire. However, ADMIXTURE modelling of Côte d’Ivoire samples did not result in a stable solution supporting a second, separate source population (Supplementary Fig. S3).

### *W. bancrofti* whole-exome sequencing and performance evaluation

In preparation for developing a targeted sequence capture panel to facilitate sequencing of mf DNA in samples with high human contamination, we first improved the annotation of the existing genome assembly. The reference genome of *W. bancrofti* was updated by Small et al.^9^ with a significant improvement in the contiguity over the previously published assembly. However, the quality of protein-coding gene annotation remained relatively poor. We re-annotated the genome to improve the accuracy of gene models. After re-annotation, the mean number of exons per gene increased from 5.3 to 9.1, and the mean CDS length increased from 0.73 kb to 1.3 kb (Table 2). The detection of full-length BUSCOs (highly-conserved core *Nematoda* genes)^58^ increased from 47.5% to 98.4%, indicating a substantial number of incomplete, fragmented or missing gene models were corrected in our updated gene set (Table 2).

**Table 2:** Genome annotation and completeness statistics (based on longest isoforms) of *W. bancrofti*, related filarial species and the model nematode *C. elegans*.

	*Wuchereria bancrofti*		*Brugia malayi*	Loa loa	*Onchocerca volvulus*	*Caenorhabditis elegans*
	Small 2019	Re-annotation				
BioProject	PRJNA275548		PRJNA10729	PRJNA37757	PRJEB513	PRJNA13758
Total genome length (bp)	88,416,250		88,235,797	91,373,458	96,427,137	100,286,401
Number of protein-coding genes	9,651	11,166	10,878	14,908	12,109	19,981
Total CDS length (bp)	7,063,809	14,859,837	14,876,705	14,767,276	14,979,205	24,568,839
Mean CDS length (bp)	732	1,331	1,368	991	1,237	1,230
% of genome covered by CDS	8	16.8	16.9	16.2	15.5	24.5
Mean exon length (bp)	144	147	160	150	199	227
Mean intron length (bp)	358	371	355	333	431	327
Mean exons per mRNA	5.3	9.1	9.1	6.6	8.8	4.6
Complete BUSCOs	47.5%	98.4%	98.9%	98.2%	98.2%	100.0%
Complete and single-copy	46.9%	96.8%	98.3%	97.9%	97.2%	99.6%
Complete and duplicated	0.5%	1.6%	0.6%	0.3%	1.1%	0.4%
Fragmented BUSCOs	6.2%	0.3%	0.2%	1.5%	0.5%	0.0%
Missing BUSCOs	46.3%	1.3%	0.9%	0.3%	1.3%	0.0%

**Fig. 8:**
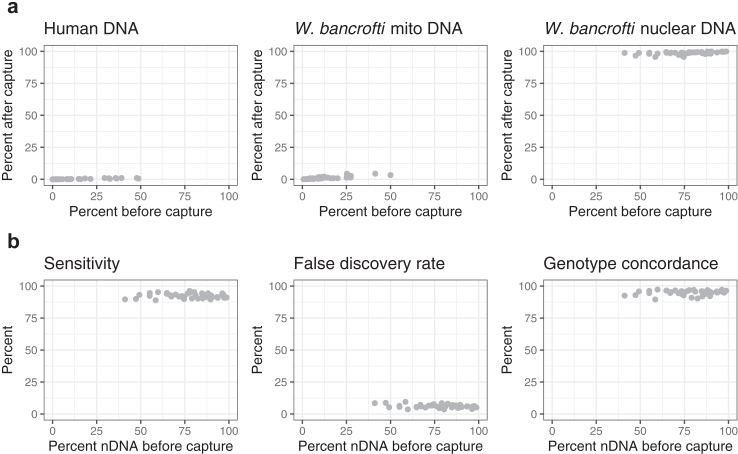
Performance assessment of *W. bancrofti* exome sequencing. (a) Reduction of human and mitochondrial DNA and enrichment of parasite nuclear DNA (nDNA) by hybridization capture of sequencing libraries. (b) Accuracy of variant calling based on ∼9 k exonic SNPs.

We designed custom hybridization capture probes (120 bp) comprised of 150,846 oligonucleotide baits targeting the coding exome of *W. bancrofti*. The design covered 13.2 Mb of 14.9 Mb total coding exome (88.7%) and 1.6 Mb of adjacent intronic/intergenic regions. Capture experiments were performed to evaluate the effectiveness of parasite nuclear genome (nDNA) enrichment using 48 single mf libraries containing a range of human DNA (up to 48.7%) and *W. bancrofti* mtDNA (up to 50.0%). On average across all samples, 95.1% of mapped bases were localized on or near (<250 bp) the regions targeted by the probes (i.e., the baited regions), indicating an overall high on-target rate. A mean coverage of 427.5x was achieved across these baited regions, representing a 128-fold enrichment above the genomic background. We compared the sequence composition of WGS and exome capture libraries derived from the same single mf source DNA (Fig. 8a). The hybridization capture approach effectively enriched *W. bancrofti* nDNA sequences. In addition, we observed a >90-fold reduction of human derived reads after exome capture among the samples that contained >5% human derived reads (n = 20). Similarly, an >18-fold reduction of mtDNA was observed among samples that initially contained >5% mtDNA (n = 28).

We called SNP variants using the exome data and assessed the accuracy of the resulting genotypes relative to the call set generated from the whole-genome data that served as the truth-set. On average across the 48 samples, ∼9 K non-reference variants were called within the baited coding regions per sample. The mean sensitivity, false discovery, and genotype concordance rates were 92.8%, 4.9% and 96.0%, respectively. We then investigated whether the pre-capture library composition significantly affected these genotype metrics (Fig. 8b). To minimize confounding due to variation in read coverage between samples, we subsampled the exome sequencing data to a uniform library size prior to variant calling. The mean sensitivity, false discovery, and genotype concordance rates of the variant call set from the subsampled exome libraries were 92.3%, 6.0% and 95.0%, respectively. Within the range tested, the genotyping quality of exome data was minimally affected by the amount of human and/or *W. bancrofti* mitochondrial DNA present in the sample (Fig. 8b).

## Discussion

This study represents a significant advance in overcoming the technical challenges that have historically impeded population-scale genomic analysis of LF-causing worms. Our initial step was to identify a method that could efficiently amplify the filarial worm genome without compromising subsequent genetic analysis. We evaluated genome coverage patterns and found that whole-genome amplification (WGA) outperformed selective whole-genome amplification (SWGA). SWGA introduced greater coverage bias than WGA, requiring four times more sequencing data than WGA to achieve similar genome coverage. This suggested that WGA would perform better than SWGA for samples with a parasite to host DNA ratio of up to 1:3, under the conservative assumption that only parasite DNA is amplified in SWGA, and that host and parasite DNA are amplified with equal efficiency in WGA. For this reason, we optimised our single mf sequencing method based on WGA. Although both WGA and SWGA have been used for filarial parasites in the past, a stringent comparison regarding their efficiency and accuracy had not been conducted.^9^^,^^59^ By quantifying genotyping accuracy in relation to the amplification method, we observed an increase in the number of false-positive variants and discordant genotypes when using either WGA or SWGA. Despite this, samples clustered in PCA based on the worm from which the DNA was isolated, rather than by the amplification method or library size. This indicates that genome-wide SNP data generated from amplified DNA can accurately capture the genetic relationships between individual worms. These comparative data will also be valuable in future studies for determining whether specific genetic analyses (such as genome-wide ROH analysis, PSMC modelling, etc.) are sensitive to genotyping errors introduced by WGA or SWGA.

Population genomic studies based on sequencing individual filarial worms that cause LF are very limited, and the analyses have mainly been based on mtDNA or a very low breadth of coverage of the nDNA.^11^^,^^60^^,^^61^ To improve nDNA coverage, our approach incorporated multiple washing steps and employed qPCR-based quality screening to ensure all samples had acceptable worm-to-human DNA ratios. Our results report analyses that cover, on average, more than 95% of the autosome, 85% of the X-chromosome, and 99% of the mtDNA with at least 10x coverage. This represents a significant improvement over previously published datasets generated using SWGA^9^ and allows for a comprehensive genome-wide variant analysis of individual mf in *W. bancrofti*.

This improvement enabled us to test and demonstrate methods for estimating the number of reproductively active adult worms in infected participants, based on the genome-wide genetic diversity among individual mf within a host. We used *B. malayi* mf of known pedigree as a guide for interpreting the *W. bancrofti* data. Using both the autosomal and X-linked SNPs, we conducted kinship analyses to reconstruct familial relationships among individual mf. These analyses provided a quantitative measure of infection burden, specifically the number of active female breeders, even when the mitochondrial genetic diversity was too low for identifying maternal lineages. Efforts to eliminate LF are guided by transmission modelling using several models.^62^, ^63^, ^64^, ^65^ However, these models are based on empirical estimates of reproductively active female worms. The ability to provide experimental data on the number of female worms both before and after treatment could greatly enhance the accuracy of these models.

Our approach enabled us to genetically track the survival and fecundity of unobserved adults throughout the treatment process, differentiating between new and persistent infections. Analysis of our Côte d’Ivoire *W. bancrofti* mf data suggested reinfection in one participant and recrudescence in three others. The fact that we discovered a participant with new infection indicates ongoing transmission, despite the low prevalence of *W. bancrofti* in the area and ongoing MDA. Non-compliance with MDA is a major problem for the Global Program to Eliminate LF and should be specifically addressed in the Côte d’Ivoire national program.^66^ In parallel, we also detected treated participants with persistent infections. This is not surprising because in Côte d’Ivoire, only about 71% of infected participants are free of mf after 12 months of IDA triple-drug treatment.^21^ It is important to note that efficacy of IDA shows geographical differences; for example, in Papua New Guinea (PNG), 96% of treated participants are free of mf 12 months after treatment.^67^ Our whole genome data show that mf from Côte d’Ivoire can be clearly differentiated from mf from PNG, and it would be possible to identify ancestry-informative markers for each strain.

Characterisation of the (unobserved) adult parasite population through kinship reconstruction of their offspring by the use of genetic analysis can be complicated by a number of factors that may also vary between species and populations.^68^ Classifying individuals into kinship categories by comparing their genotypes is challenging because genetic relatedness—the proportion of the genome shared between two individuals by descent from a common ancestor—is a continuous parameter. Its variance depends on the number of chromosomes and their crossover rates,^69^ and it may not correspond to theoretical expectations based on pedigree relationships, especially when founders are related and not outbred, or they are from very different populations.^56^^,^^70^^,^^71^ Unlike some helminth species where overlapping generations can occur in a host, for example, due to autoinfections, the intrapopulation of *W. bancrofti* mf in a human host can be expected to mainly contain groups of full and/or half-siblings. We therefore employed a sibship reconstruction approach and temporally tracked the sibling groups to make inferences about the number and survival/fecundity of the adult breeders through the treatment process. This approach is likely more accurate than simply evaluating individual dyads between pre- and post-treatment samples because it considers the relationships among all genotypes simultaneously.^72^ Successful sibship reconstruction, however, will depend on the number of individuals sampled per family; therefore, this approach is not well-suited for species that generally result in infections with a high adult worm burden or for species with extreme reproductive skew among the breeders.^20^ The complexity of the expected kinship structure is another important factor. Only full-sibling inference is required to estimate the number of breeding adults in species with a monogamous mating system, whereas half-sib groups need to be determined in polygamous species, such as filarial nematodes.^73^ To reliably determine maternal sibling families in *W. bancrofti*, we utilised X-linked haplotype information in addition to autosomal relatedness. Although the maternally inherited mitochondrial haplotypes were not used in our analysis due to insufficient genetic diversity among the samples, they can be informative markers in other species and/or populations.^74^ Another important caveat of our approach is the possibility of erroneously concluding that there is a new infection in longitudinal analysis due to insufficient sampling of mf at the earlier time point. Likewise, insufficient sampling of mf at the later time point may result in a false negative result. Additionally, our approach cannot detect infections during the prepatent period, which is the interval between the entry of the L3 larvae and the appearance of detectable mf in the host. This period can last several months in filariasis. Therefore, temporal sampling should be carefully designed to meet the requirements of specific research goals.

Population structure analysis that included samples from Haiti, Kenya, Mali, and PNG indicated that *W. bancrofti* in Côte d’Ivoire formed a distinct population that could be differentiated from parasites collected in other countries, including West African samples from Mali, despite their relative geographical proximity. Because of the sparse geographical sampling in the currently available dataset, it is not clear if *W. bancrofti* will display patterns of fine-scale genetic structure at shorter distances. However, we anticipate that as the database of geographically diverse *W. bancrofti* samples expands in the future, it may become possible to determine the genetic ancestry of samples, identify the source population, and spatially track parasite movement associated with host/vector migration, as has been exemplified in schistosomiasis control efforts.^6^ Furthermore, our analysis helped identify mf that are the progeny of parasites that have resumed reproduction after IDA treatment (e.g., those in participants 149 and 155), as well as progeny from parasites that either did not survive or ceased reproduction after treatment (e.g., pre-treatment mf in participant 111). This lays the groundwork for future genome-wide association analyses (once a sufficient sample size is reached) to understand the genetic factors that influence variations in parasite drug response and to identify genetic markers for surveillance.

Finally, we incorporated multiple layers of quality control steps into our WGA pipeline to exclude samples with high levels of host contamination and/or DNA degradation. Residual human DNA in samples can result in lower worm genome coverage, and small circular genomes (e.g., mitochondrial genome) can be preferentially amplified during WGA when the input DNA is fragmented. However, samples from field isolates are frequently suboptimal due to storage and/or shipping conditions. To facilitate the use of most clinical field samples, we developed a whole-exome capture panel that enriches for *W. bancrofti* nDNA from a mixed sample containing host DNA. While several *W. bancrofti* genome assemblies with increasing contiguity have been published,^9^^,^^10^^,^^75^ the quality of the gene models was insufficient for designing high-quality exon capture probes. Therefore, we re-annotated the *W. bancrofti* genome and improved the BUSCO completeness score to 98.4%. By systematically comparing library compositions and genotyping accuracies, we demonstrated that a hybridisation capture approach can be employed to achieve unbiased sampling of mf without the need to exclude samples with high levels of human DNA contamination and/or those of suboptimal quality. In the future, we anticipate that targeted genotyping approaches such as hybridization capture or multiplex PCR-based methods can be designed to focus on a smaller set of “informative” SNPs for sibship inference and parentage analysis. These methods would offer improved throughput and cost-effectiveness. Developing a well-curated database of genetic variations in clinical samples will then be crucial for understanding changes in parasite populations following drug treatment and interventions. Such a database, encompassing both global and regional genetic variation, will support the identification of possible source populations in cases of reinfection. Additionally, given a large enough sample size, the database could facilitate a genome-wide association study (GWAS) to investigate the contribution of parasite genetics to treatment outcomes.

Using a combination of laboratory-produced *B. malayi* samples and field-collected *W. bancrofti* samples, we showed that whole-genome amplification is more effective and accurate than selective whole-genome amplification for the genomic analysis of mf. We provide evidence that analysing mitochondrial genomes is insufficient for differentiating persistent infections from newly acquired ones; however, this differentiation can be reliably achieved through the analysis of the nuclear genome. Targeted exon capture is a robust approach for enriching the coding sequences of filarial parasites from lower-quality DNA samples. Our study lays the groundwork for developing molecular genetic tools to estimate worm burdens and monitor parasite populations, which will be invaluable for the successful implementation of mass drug administration programs aimed at eliminating lymphatic filariasis.

## Contributors

Conceived and designed the study: PUF and MM.

Collected specimens: MA and BGK.

Biological and sequence data database maintenance: YC.

Sample preparation, extraction of DNA, WGA and SWGA and qPCR: KF.

Performed data analysis: YC.

Interpreted the data: YC, PUF and MM.

Wrote the paper: YC, PUF, KF and MM.

All authors read and approved the final version of the manuscript.

Young-Jun Choi and Makedonka Mitreva have accessed and verified the underlying data.

## Data sharing statement

The dataset supporting the conclusions of this article is available in the NCBI Sequence Read Archive (SRA, https://www.ncbi.nlm.nih.gov/sra) under the study accession number SRP463861. Sample metadata is available in Supplementary Table S10. The exome capture reagents are available from Twist Bioscience with design ID TE-92630402. The new protein-coding gene annotations for *W. bancrofti* (PRJNA275548) are available in WormBase Parasite (WBPS19). The command-line arguments used in the analysis are described in Supplementary Text S1.

## Declaration of interests

The authors have no competing interests to disclose.
