# Supplementary material for: Distinguishing recrudescence from reinfection in lymphatic filariasis
Source: eBioMedicine. 2024 Jun 7;105:105188. doi: 10.1016/j.ebiom.2024.105188 (PMC11200287; doi:10.1016/j.ebiom.2024.105188)
Supplement: Supplementary Figs. S1–S3 [file mmc1.pdf]

Supplementary Fig. S1 Cluster dendrogram displaying autosomal relatedness of *Wuchereria bancrofti* microfilariae based on hierarchical clustering of kinship coefficients. Sample IDs consist of three distinct pieces of information connected by underscores: microfilaria ID, KING family membership, and maternal sibling group membership. "NA" indicates not available, and "ND" indicates not determined.

Participant ID: 111

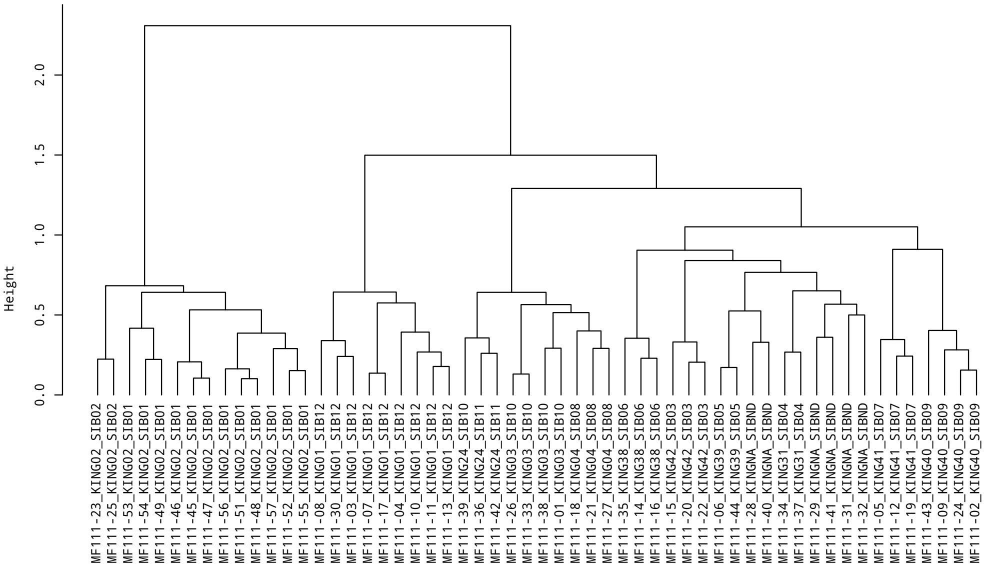

Participant ID: 149

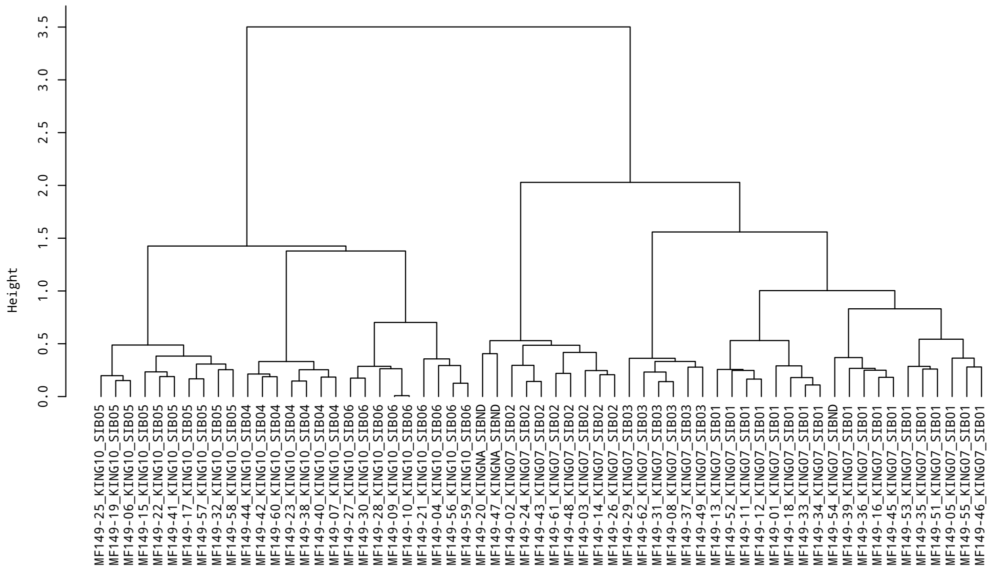

Participant ID: 154

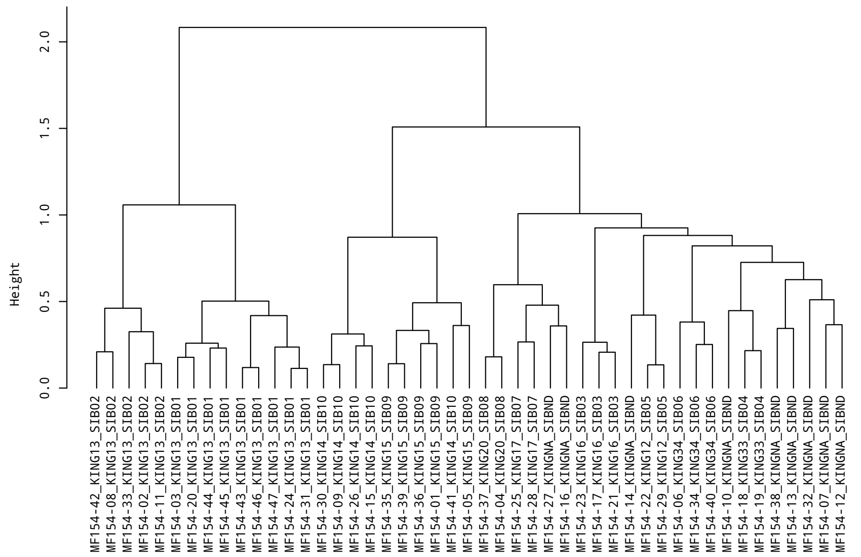

Participant ID: 155

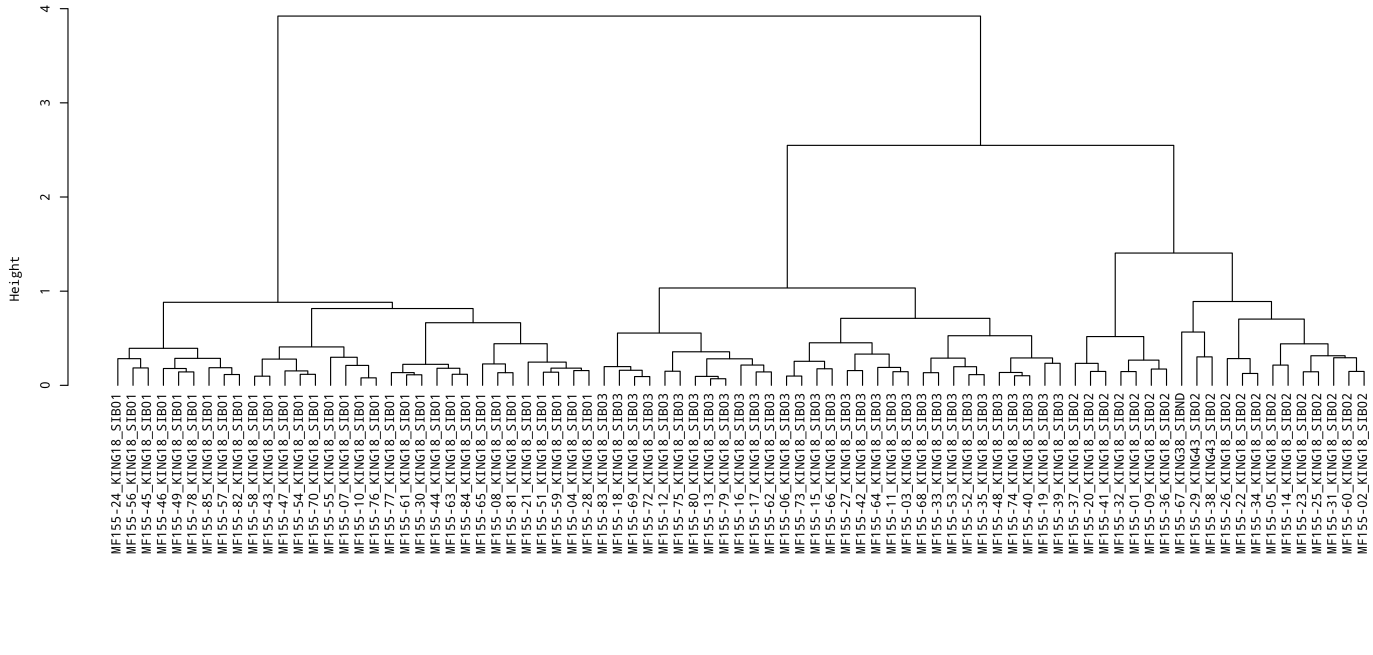

Supplementary Fig. S2 Maximum-likelihood phylogenetic trees of the X-linked haplotypes in *Wuchereria bancrofti* male microfilariae. Sample IDs consist of two distinct pieces of information connected by underscores: microfilaria ID and haplotype ID. "ND" indicates not determined.

Participant ID: 111

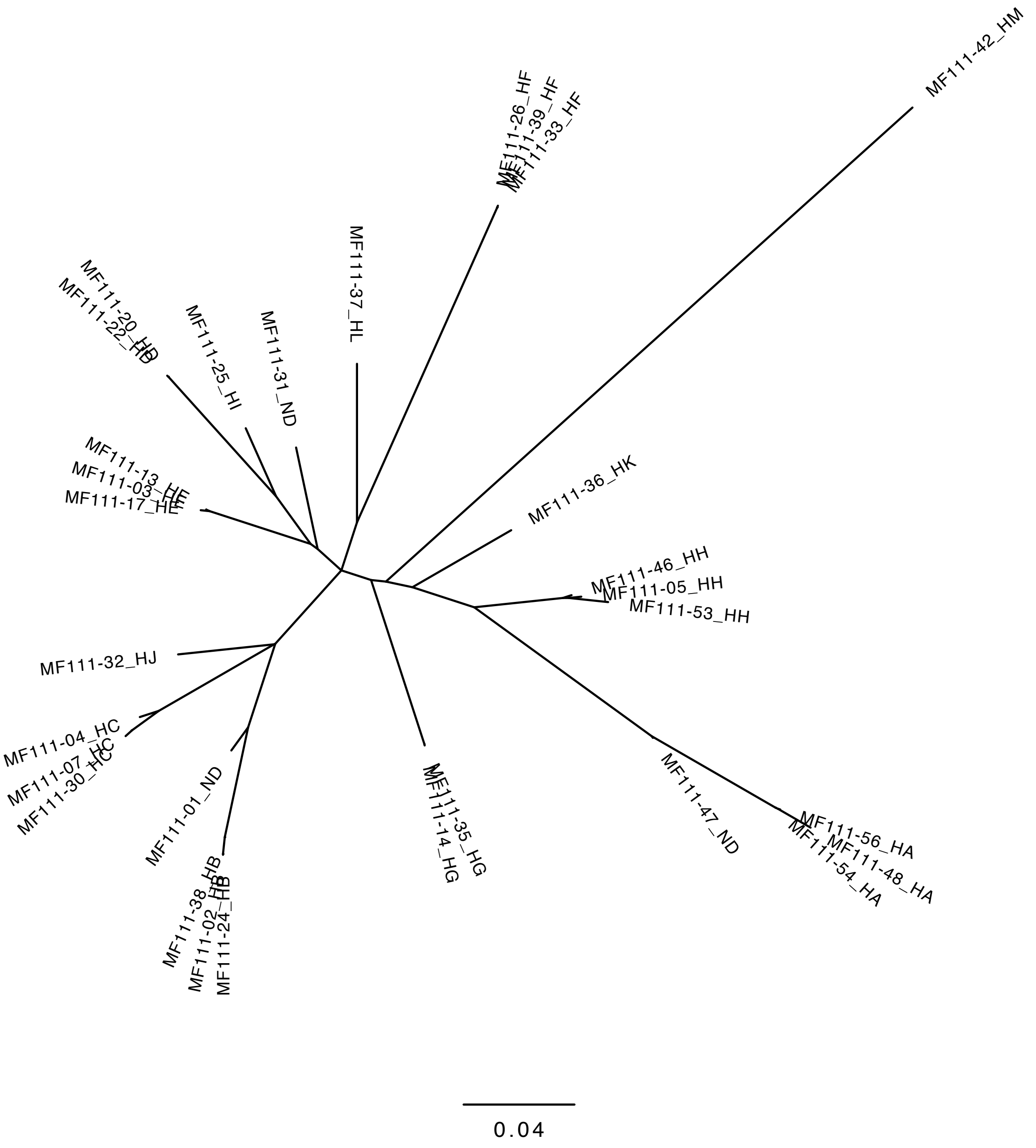

Participant ID: 149

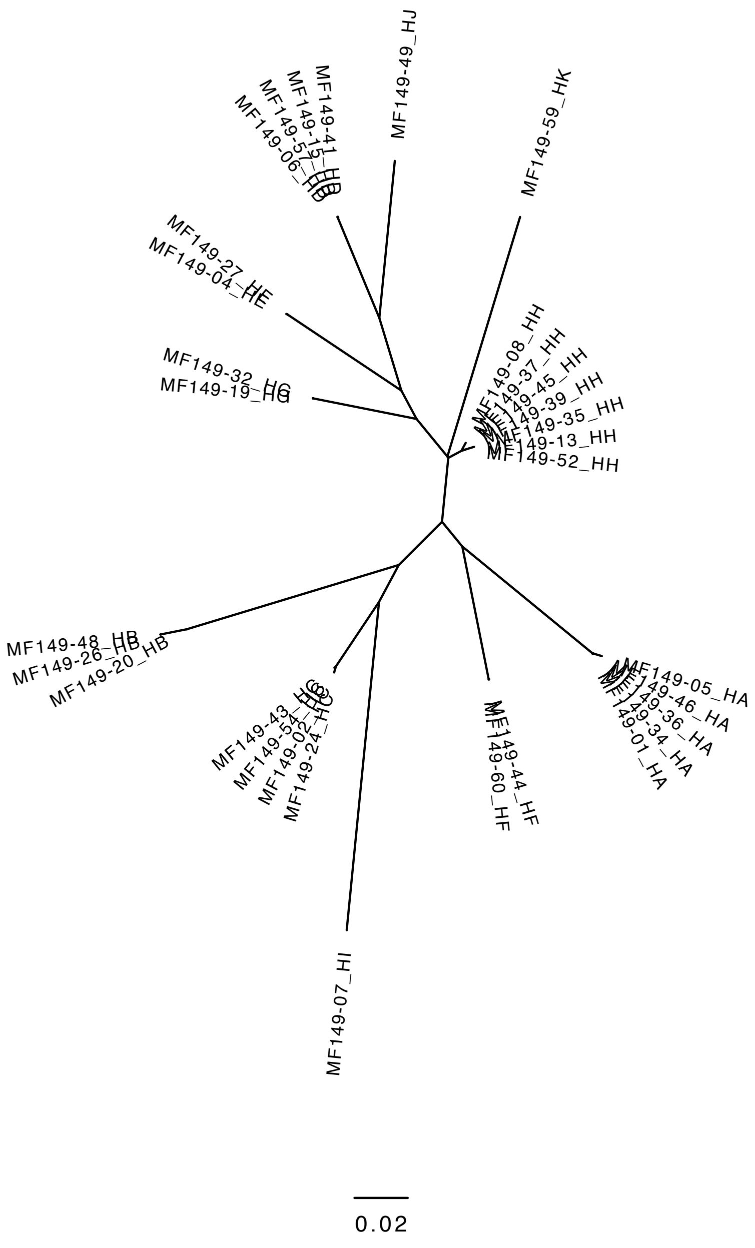

Participant ID: 154

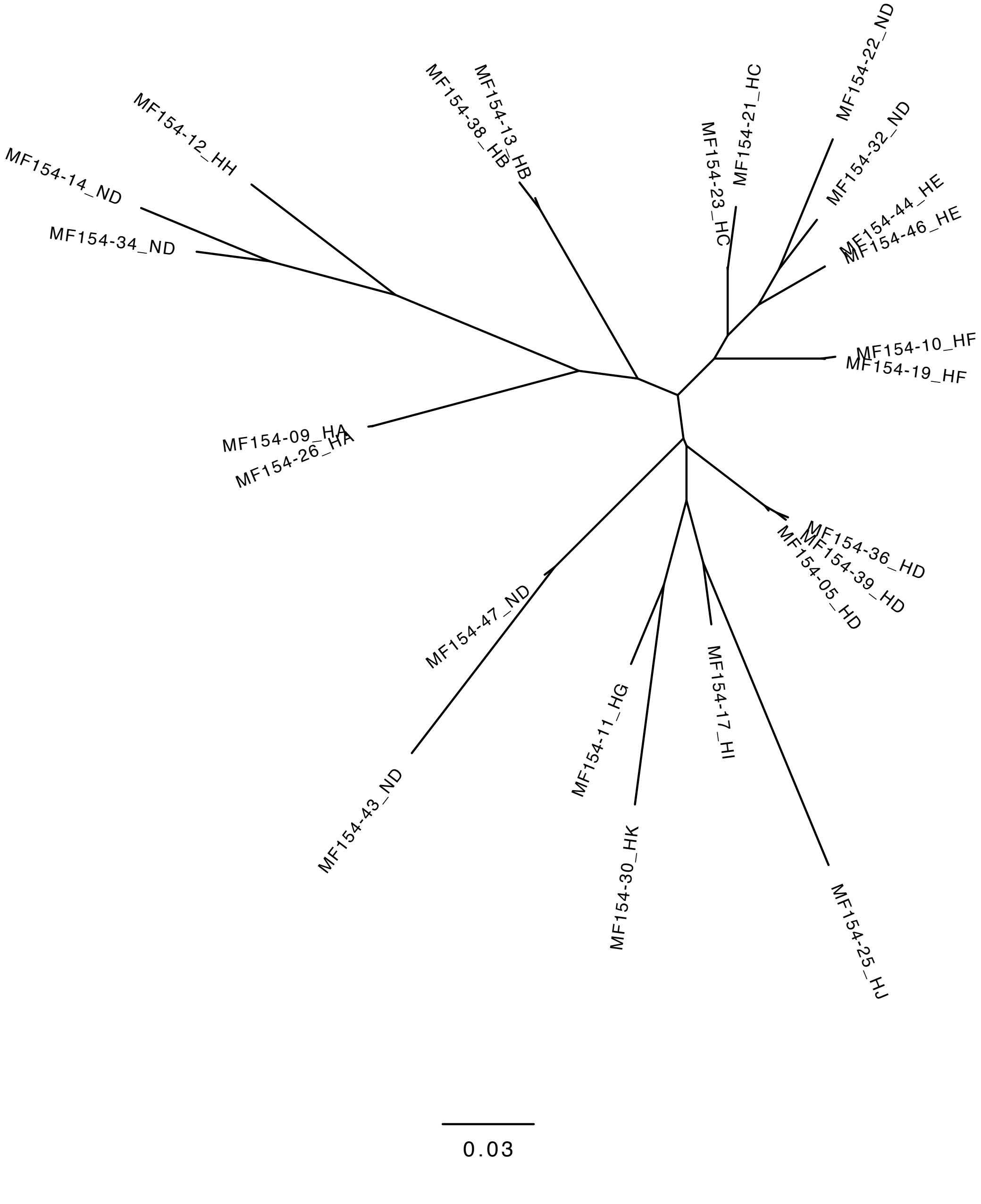

Participant ID: 155

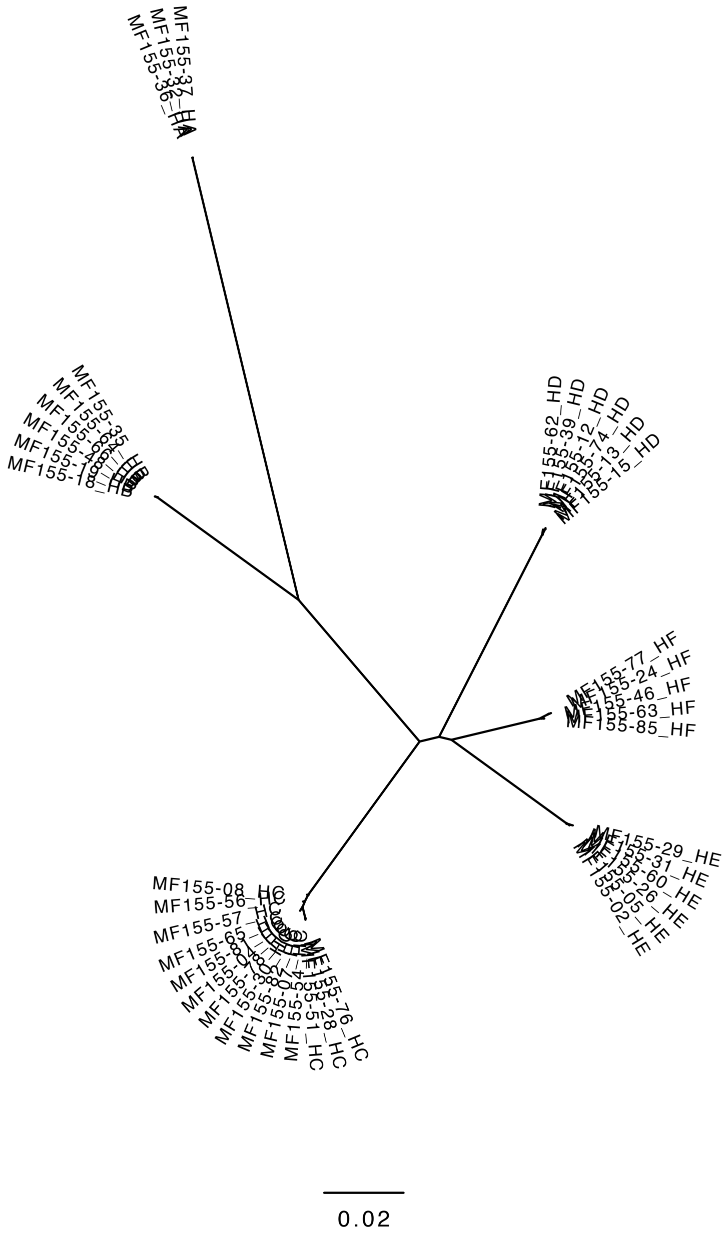

Supplementary Fig. S3 Admixture modelling of *Wuchereria bancrofti* genetic diversity in Côte d'Ivoire. (a) The major mode from 100 ADMIXTURE runs was displayed, and the stability of the solution was reported. Analysis was based on 2,380 LD-pruned autosomal SNPs and 55 unrelated Côte d'Ivoire samples. (b) All 100 solutions found by ADMIXTURE for K = 2.

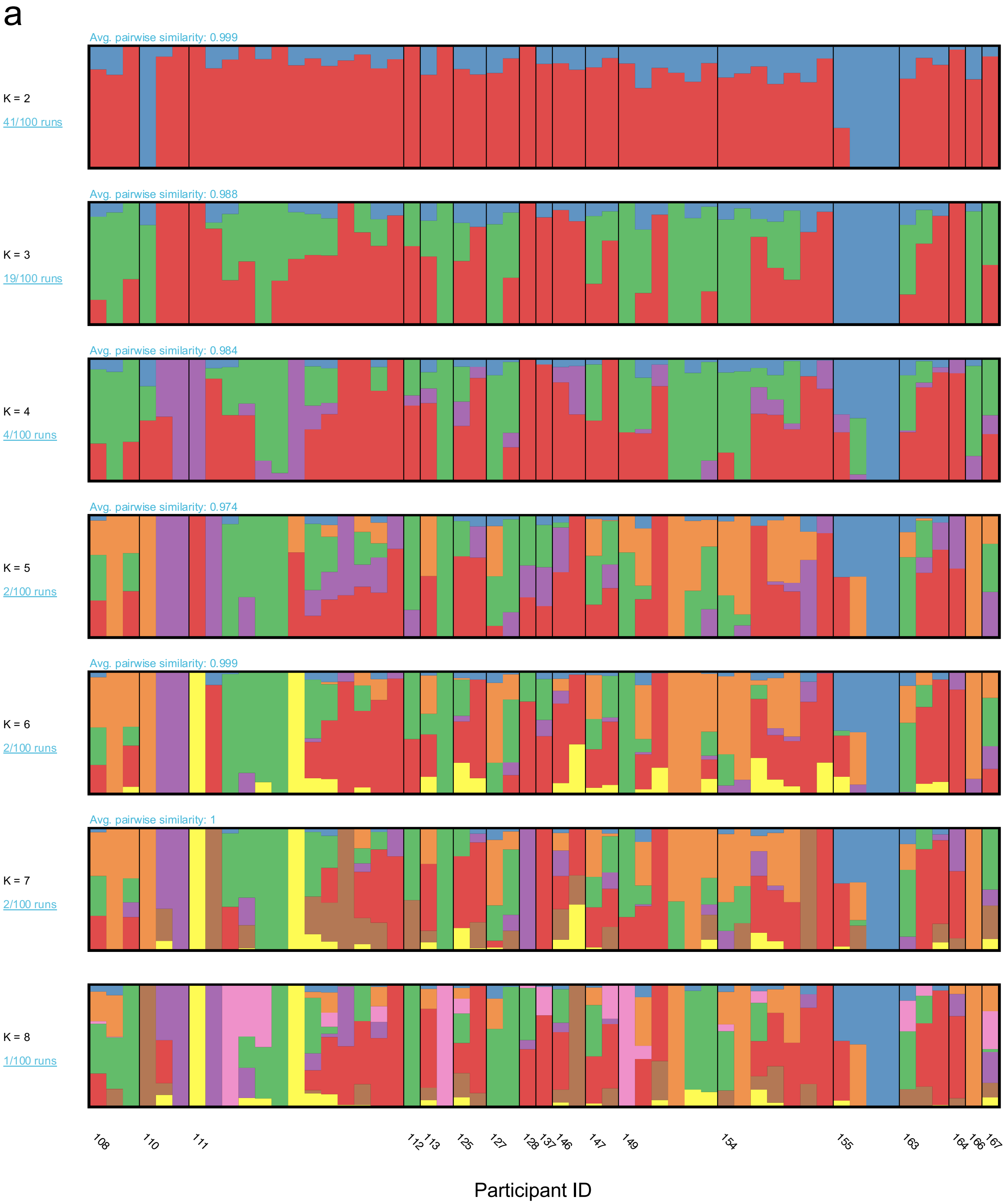

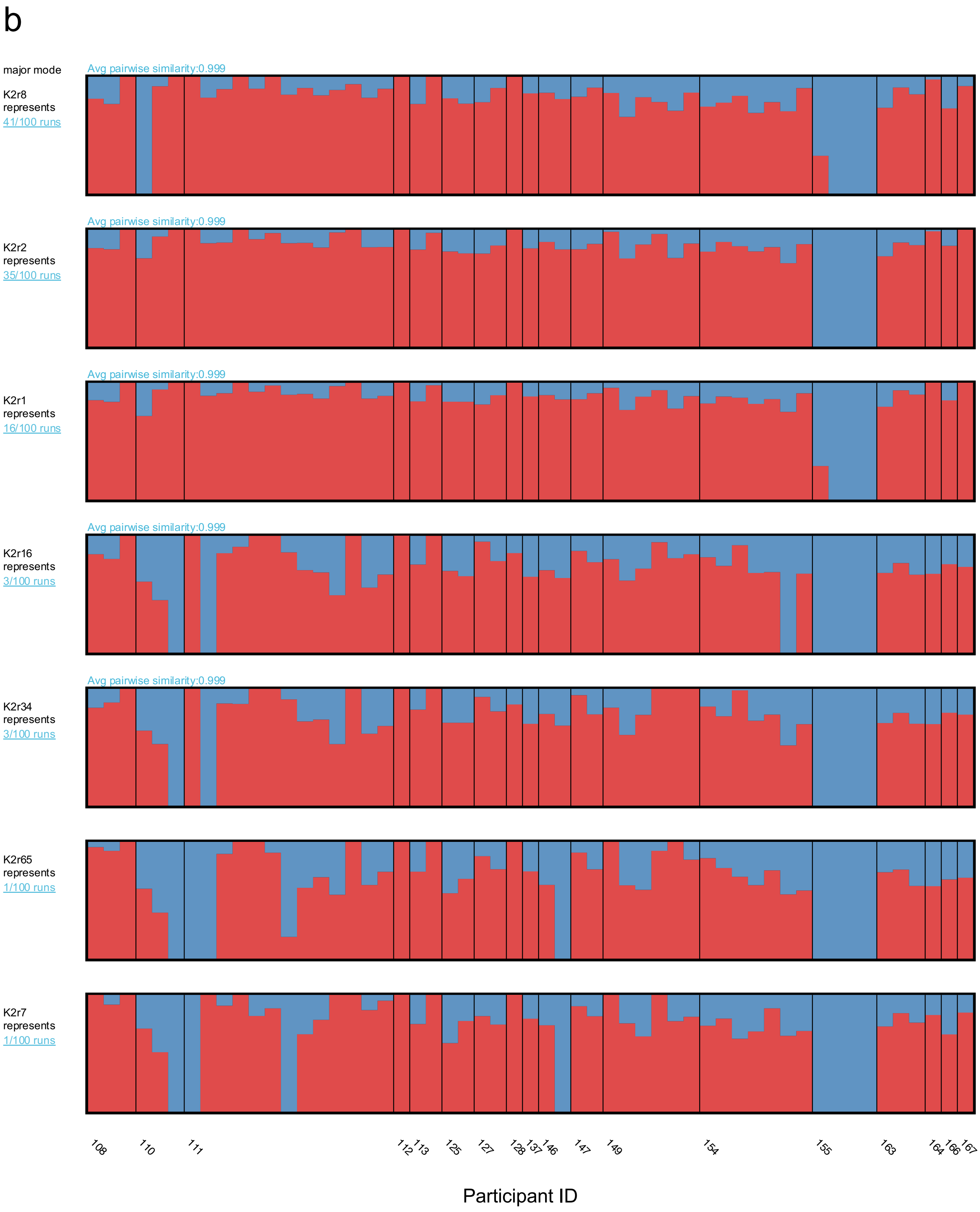
b
major mode
Avg pairwise similarity:0.999
K2r8
represents
41/100 runs
Avg pairwise similarity:0.999
K2r2
represents
35/100 runs
Avg pairwise similarity:0.999
K2r1
represents
16/100 runs
Avg pairwise similarity:0.999
K2r16
represents
3/100 runs
Avg pairwise similarity:0.999
K2r34
represents
3/100 runs
K2r65
represents
1/100 runs
K2r7
represents
1/100 runs
108
110
111
112
113
125
127
128
137
146
147
149
154
155
163
164
166
167
Participant ID
