# Supplementary material for: Distinguishing recrudescence from reinfection in lymphatic filariasis
Source: eBioMedicine. 2024 Jun 7;105:105188. doi: 10.1016/j.ebiom.2024.105188 (PMC11200287; doi:10.1016/j.ebiom.2024.105188)
Supplement: Supplementary Text S1 [file mmc3.pdf]

**Supplementary Text S1.** The command-line arguments used in the analysis.

## Adapter/quality trimming of reads, reference alignment, and variant calling

```
# Adapter/quality trimming of reads with trimmomatic-0.39

java -jar trimmomatic-0.39.jar PE -threads <cpu#> <Read1.fq.gz> <Read2.fq.gz> <forward_paired.fq.gz> <forward_unpaired.fq.gz> <reverse_paired.fq.gz>
<reverse_unpaired.fq.gz> ILLUMINACLIP:<adapters.fa>:2:30:10:2 LEADING:3 TRAILING:3 SLIDINGWINDOW:4:15 MINLEN:36
```

```
# Reference alignment with bwa-0.7.17

bwa index <reference_genome.fa>

bwa mem -t <cpu#> -M -o <alignment.sam> <reference_genome.fa> <forward_paired.fq.gz> <reverse_paired.fq.gz>
```

```
# Converting SAM to BAM and adding Read Group information with picard-2.26.2

java -jar picard.jar AddOrReplaceReadGroups I=<alignment.sam> O=<alignment.bam> RGID=<RGID> RGLB=<RGLB> RGPL=ILLUMINA RGSM=<RGSM> RGPU=<RGPU>
SORT_ORDER=coordinate CREATE_INDEX=True
```

```
# Merging BAM files per biological sample with samtools-1.13

samtools merge -o <sample.bam> -b <bam.list>

samtools index <sample.bam>
```

```
# Generating subsampled BAM files

samtools view -bs <fraction> <input.bam> > <subsampled.bam>
```

```
# Generating genome coverage statistics (mapping quality >10 and read depth >10x)

samtools view -q 10 -b <sample.bam> > <sample.q10.bam>

samtools stats -t <target-regions> -g 10 <sample.q10.bam>
```

```
# Variant calling and filtering with gatk-4.2.2.0

gatk MarkDuplicates --REMOVE_DUPLICATES true --CREATE_INDEX true --METRICS_FILE <dedup.metrics> --INPUT <sample.bam> --OUTPUT <sample.dedup.bam> --
OPTICAL_DUPLICATE_PIXEL_DISTANCE 2500

gatk HaplotypeCaller --reference <reference_genome.fa> -I <sample.dedup.bam> -O <sample.ploidy_2.g.vcf.gz> -ERC GVCF -ploidy 2

gatk GenomicsDBImport --genomicsdb-workspace-path GenomicsDB --reference <reference_genome.fa> --sample-name-map <cohort.sample_map> --intervals
<interval_list>

gatk GenotypeGVCFs -V gendb://GenomicsDB --reference <reference_genome.fa> --intervals <interval_list> -O <output.vcf.gz>

gatk SelectVariants -V <output.vcf.gz> -O <SNP.vcf.gz> -select-type SNP

gatk VariantFiltration -V <SNP.vcf.gz> -O <SNP.filtered.vcf.gz> -filter "QD < 2.0" --filter-name "QD2" -filter "QUAL < 30.0" --filter-name "QUAL30" -
filter "SOR > 3.0" --filter-name "SOR3" -filter "FS > 60.0" --filter-name "FS60" -filter "MQ < 40.0" --filter-name "MQ40" -filter "MQRankSum < -12.5"
--filter-name "MQRankSum-12.5" -filter "ReadPosRankSum < -8.0" --filter-name "ReadPosRankSum-8" -filter "DP > <median_depth*2>" --filter-name "DP"
```

```
# Genotype concordance analysis

java -jar picard.jar GenotypeConcordance CALL_VCF=<VCF> CALL_SAMPLE=<sample_ID> O=<out_file> TRUTH_VCF=<VCF> TRUTH_SAMPLE=<sample_ID>
INTERVALS=<interval_list>
```

```
# Exome sequencing statistics

java -jar picard.jar CollectHsMetrics I=<sample.bam> O=<hs_metrics.txt> R=<reference_genome.fa> BAIT_INTERVALS=<probes.interval_list>
TARGET_INTERVALS=<target.interval_list>
```

## Population structure analysis using PCA

```
# Converting VCF to BCF files after filtering variants with bcftools-1.15

bcftools view --samples-file <sample.list> -f "PASS" --targets-file <autosome.bed> <SNP.filtered.vcf.gz> --min-ac 4:minor --include 'F_MISSING<0.05'
--min-alleles 2 --max-alleles 2 | bcftools convert -O b -o <autosome.bcf>

bcftools index <autosome.bcf>
```

```
# Identifying a set samples that are not closely related using akt-0.3.3

akt kin -M 1 --force <autosome.bcf> > <kinship.txt>

akt unrelated <kinship.txt> > <unrelated.ids>
```

```
# Performing PCA on unrelated samples after LD-based SNP pruning with plink-1.90

plink --vcf <SNP.filtered.vcf.gz> --vcf-filter --allow-extra-chr --double-id --extract <autosome.bed> --range --out <unrelated> --mac 4 --geno 0.05 -
-keep <unrelated.list> --indep-pairwise 500kb 1 0.2 --set-missing-var-ids @:# --biallelic-only
```

```
plink --vcf <SNP.filtered.vcf.gz> --vcf-filter --allow-extra-chr --double-id --extract <unrelated.prune.in> --out <unrelated> --pca --keep
<unrelated.list> --set-missing-var-ids @:#
```

## Population structure analysis using ADMIXTURE

```
# Converting VCF to PLINK BED files with plink-1.90

plink --vcf <SNP.filtered.vcf.gz> --vcf-filter --allow-extra-chr --double-id --extract <unrelated.prune.in> --out <unrelated> --make-bed –keep
<unrelated.list> --set-missing-var-ids @:#


# Run admixture-1.3.0 for different values of K, each with 100 runs (Lie et al., 2020; PMID: 31975164)

prefix=unrelated

for r in {1..100}; do for K in {2..8};
do
admixture -s ${RANDOM} ${prefix}.bed $K
mv ${prefix}.${K}.Q ${prefix}.K${K}r${r}.Q
done; done


# Creating Qmap file for pong

createQmap(){
local r=$1
local K=$2
awk -v K=$K -v r=$r -v file=${prefix}.K${K}r${r} 'BEGIN{printf("K%dr%d\t%d\t%s.Q\n",K,r,K,file)
}' >> ${prefix}.multiplerun.Qfilemap
}
export -f createQmap
for K in {2..8}; do for r in {1..100}; do createQmap $r $K; done; done


# Runing pong-1.5

pong -m $prefix.multiplerun.Qfilemap --greedy -i <Ind2pop.txt> -n <pop_order.txt>
```

## Maternal sibship inference

```
# Variant filtering with plink-1.90

plink --vcf <SNP.filtered.vcf.gz> --vcf-filter --allow-extra-chr --double-id --make-bed --set-missing-var-ids @:# --keep <sample.list> --extract
<autosomes.bed> --range --geno 0.05 --mac 4 --out <pop>


# Identifying potential full sibling groups with king-2.3.2

king -b <pop.bed> --kinship --prefix <pop>

king -b <pop.bed> --build --prefix <pop>

king -b <pop.bed> --cluster --prefix <pop>


# Constructing maximum-likelihood phylogenetic trees using X-linked SNPs in male microfilariae

# Variant filtering with bcftools-1.15

bcftools view --samples-file <male.list> --targets-file <X-chr_non_PAR.bed> -f "PASS" <SNP.filtered.vcf.gz> | bcftools view -o <X-chr_male.vcf.gz> -g
^het --min-ac 4:minor -

bcftools index -t <X-chr_male.vcf.gz>


# Converting VCF to TAB file with vcftools-0.1.16

zcat <X-chr_male.vcf.gz> | path-to/vcftools-0.1.16/src/perl/vcf-to-tab | grep -v '*' | sed 's|\./.|N/N|g' > <X-chr_male.tab>


# Converting TAB file to FASTA using https://code.google.com/archive/p/vcf-tab-to-fasta/

perl vcf_tab_to_fasta_alignment.pl -i <X-chr_male.tab> > <X-chr_male.fasta>


# Subsetting FASTA file to include sequences from an individual person, and converting it to PHYLIP format with trimal-1.4

samtools faidx -r <sample.list> -o <person.fasta> <X-chr_male.fasta>

trimal -in <person.fasta> -out <person.phy> -phylip


# Constructing maximum-likelihood phylogenetic trees with IQ-TREE v2.2.0

iqtree2 -s <person.phy>


# Grouping of leaves into haplotypes with TreeCluster-1.0.4

TreeCluster.py -i <person.phy.treefile> -o <person.phy.treefile.cluster> -t <threshold> -m max


# Hierarchical clustering of samples based on autosomal relatedness

# Subsetting PLINK BED file to include microfilariae from an individual person with pink-1.90
```

```
plink --bfile <pop.bed> --make-bed --out <person.bed> --keep <sample.list>


# Constructing kinship coefficient matrix with plink-2.0

plink2 --bfile <person.bed> --make-king square --out <person>


# Hierarchical clustering of samples using the PLINK output files in R

df <- read.table("person.king", header=F, sep = "\t")
id <- read.table("person.king.id", header=F, sep = "\t")
rownames(df) <- id$V1
colnames(df) <- id$V1
df_zero <- pmax(as.matrix(df),0) # Convert negative coefficient values to zero
df_dist <- 0.5 - df_zero # Convert similarity to dissimilarity
hc <- hclust(as.dist(df_dist), method = 'ward.D')
plot(hc, hang = -1)
```

## Mitochondria haplotype analysis

```
# No depth filter applied during GATK SNP filtering

gatk VariantFiltration -V <SNP.vcf.gz> -O <SNP.filtered.vcf.gz> -filter "QD < 2.0" --filter-name "QD2" -filter "QUAL < 30.0" --filter-name "QUAL30" -
filter "SOR > 3.0" --filter-name "SOR3" -filter "FS > 60.0" --filter-name "FS60" -filter "MQ < 40.0" --filter-name "MQ40" -filter "MQRankSum < -12.5"
--filter-name "MQRankSum-12.5" -filter "ReadPosRankSum < -8.0" --filter-name "ReadPosRankSum-8"

bcftools view --samples-file <sample.list> --targets 'gi|346421827|gb|JF775522.1|' -f "PASS" <SNP.filtered.vcf.gz> | bcftools view -o <mito.vcf.gz> -
g ^het --min-ac 4:minor –

bcftools index -t <mito.vcf.gz>


# Converting VCF to TAB file with vcftools-0.1.16

zcat <mito.vcf.gz> | path-to/vcftools-0.1.16/src/perl/vcf-to-tab | sed 's|\./.|N/N|g' > <mito.tab>


# Converting TAB file to FASTA using https://code.google.com/archive/p/vcf-tab-to-fasta/

perl vcf_tab_to_fasta_alignment.pl -i <mito.tab> | sed -e '/^[^>]/s/[^ATGCatgc]/-/g' <mito.fasta>


# Converting FASTA to PHYLIP format with trimal-1.4

trimal -in <mito.fasta> -out <mito.nex> -nexus -nogaps


# PopART-1.7 (GUI) was used for haplotype network construction.
```
